# Multicenter, cluster-based, superiority trial of a multicomponent lifestyle intervention versus usual care for reducing cardiometabolic risk in individuals with psychotic disorders over 36 months: the LAGOM protocol

**DOI:** 10.1186/s12888-026-08315-3

**Published:** 2026-06-23

**Authors:** Hemen Najar, Erik Jedenius, Andreas Fröberg, Anna Olofsson, Caroline Holmbom, Cathrin Kölborg Björlin, Christina Hagberg, Elin Saari-Bladmyr, Emelie Hantelius, Erik Wålinder, Eva Andreasson, Joacim Gustafsson, Lennart Lundin, Lina Klysing, Louise Rundqvist, Nadja Körvek, Pia Rydell, Sandra Heder, Zangin Zeebari, Christopher Holmberg

**Affiliations:** 1 Department of Psychotic Disorders, Sahlgrenska University Hospital, Gothenburg, Region Västra Götaland Sweden; 2 Institute of Neuroscience and Physiology, Section of Psychiatry and Neurochemistry, Sahlgrenska Academy, University of Gothenburg, Gothenburg, Sweden; 3 Department of Clinical Sciences Lund/Clinical Sciences Helsingborg, Lund University, Helsingborg, Sweden; 4 Swedish Schizophrenia Association, Gothenburg, Sweden; 5 Gothia Forum, Sahlgrenska University Hospital, Region Västra Götaland, Gothenburg, Sweden; 6 Jönköping International Business School, Jönköping University, Jönköping, Sweden; 7 Department of Global Public Health, Karolinska Institutet, Stockholm, Sweden; 8 Institute of Health and Care Sciences, Sahlgrenska Academy, University of Gothenburg, Gothenburg, Sweden

**Keywords:** Psychotic disorders, Cardiometabolic risk factors, Behavior change intervention, Multicomponent intervention, Integrated health care, Pragmatic clinical trial, Metabolic syndrome, Cardiovascular disease prevention, Quality of life, Cost-effectiveness

## Abstract

**Background:**

Cardiometabolic conditions—including cardiovascular disease, type 2 diabetes, and obesity—are highly prevalent among individuals with psychotic disorders. These conditions contribute substantially to reduced life expectancy, diminished quality of life, and increased societal and economic burdens. Thus, effective, individualized interventions are urgently needed. Outpatient psychiatric clinics offer an ideal setting for such efforts owing to regular patient contact and access to multidisciplinary care. We have developed a comprehensive, clinically integrated trial aimed at improving cardiometabolic health, promoting healthier lifestyles, and enhancing quality of life for individuals with psychotic disorders receiving care in the Greater Gothenburg region.

**Methods:**

LAGOM is a multicenter, naturalistic, quasi-experimental case‒control trial conducted across six geographically separate outpatient psychosis clinics within the Department of Psychotic Disorders at Sahlgrenska University Hospital, a multi-site university hospital in the Greater Gothenburg region. A total of 650 adults with psychotic disorders will be recruited from these clinics. Two clinics will implement the LAGOM intervention, whereas four will serve as control sites delivering usual care. The intervention is embedded within routine psychiatric care and grounded in behavioral science. It includes comprehensive cardiometabolic risk assessments, two visual motivational tools (QRISK3 and a body composition analyzer), personalized follow-up plans, risk-oriented referrals to primary care, and structured education for patients, relatives, and staff. The intervention is designed to be scalable, sustainable, and tailored to individual patient needs.

**Discussion:**

If proven superior to usual care, this pragmatic, multicomponent intervention—delivered within routine psychiatric care—could improve cardiometabolic health and quality of life for individuals with psychotic disorders. Embedding the intervention within existing clinical structures enhances its scalability and feasibility and, if effective, could serve as a model for wider implementation.

**Trial registration:**

ClinicalTrials.gov (NCT06781801; date registered: 16 January 2025).

**Trial status:**

Recruitment started on 27 February 2025 and will be completed on 31 December 2026. The current clinical investigation plan version is 3.1, dated 21 October 2025.

**Supplementary Information:**

The online version contains supplementary material available at 10.1186/s12888-026-08315-3.

## Background

Individuals with schizophrenia and schizophrenia-spectrum disorders (psychotic disorders) face significantly elevated risks of cardiometabolic diseases—including cardiovascular diseases (CVDs), obesity, and type 2 diabetes mellitus—compared with the general population [1]. These conditions contribute to markedly reduced life expectancy; in Sweden, people with psychotic disorders die, on average, 20 years earlier than the general population does [2]. In addition to premature mortality, these diseases negatively impact quality of life [3] and impose substantial economic burdens due to increased healthcare costs and reduced productivity [4].

Multiple factors contribute to this heightened cardiometabolic risk. These include the inherent nature of the illness, genetic predispositions, the metabolic side effects of psychotropic medications, and unhealthy lifestyle behaviors [5]. Additionally, disparities in healthcare access and inadequate somatic care further exacerbate cardiometabolic vulnerability in this population [5, 6].

In addition to these risks, both individual- and system-level barriers limit the effectiveness of lifestyle interventions and preventive care. At the individual level, cognitive impairments, persistent psychiatric symptoms, and side effects of psychotropic medication hinder the initiation and maintenance of healthy behaviors [7]. At the system level, healthcare professionals frequently rely on brief, generic lifestyle advice that fails to account for the cognitive and functional limitations of this group and consequently imposes expectations equivalent to those of the general population. The Swedish National Board of Health and Welfare has highlighted these shortcomings, calling for more individualized and sustained support to promote effective lifestyle changes [8].

Structural gaps in psychiatric care further limit progress. Although annual physical health checks are recommended in psychiatry, clear guidelines for their implementation are lacking. Knowledge among psychiatrists and other healthcare professionals regarding how to assess cardiometabolic risk factors and lifestyle habits—the core purpose of these checks—varies significantly [6, 9, 10]. Moreover, standardized follow-up routines are lacking or inconsistently applied to ensure that identified risks lead to appropriate interventions [9]. This shortcoming—particularly regarding the need for closer follow-up in patients with known risk factors such as weight gain, hereditary CVD, or diabetes—was highlighted by the Swedish National Board of Health and Welfare in its 2022 national evaluation of care and support for schizophrenia and schizophrenia-spectrum disorders [11]. Another gap is the lack of educational programs for both individuals with psychotic disorders and healthcare professionals addressing the interactions among psychosis, lifestyle, and cardiometabolic health. Finally, collaboration between psychiatry and primary care—particularly regarding the identification and follow-up of cardiometabolic risks—needs to be strengthened to improve overall health outcomes in this population.

Some clinical trials have demonstrated the potential benefits of tailored interventions in improving cardiometabolic outcomes for people with psychotic disorders. For example, the ACHIEVE trial, an 18-month behavioral weight loss intervention, demonstrated weight loss [12]. Similarly, two 30-month programs—one health-promoting and the other individualized and health-oriented—reported reductions in CVD risk [13] and diabetes incidence [14]. However, most previous interventions have been constrained by a range of limitations that affect their clinical effectiveness, generalizability, and feasibility in routine psychiatric care. Many were short-term, typically lasting a year or less [15–19], which may not allow sufficient time to achieve meaningful improvements—aside from exceptions such as the CRESSOB study [20]. Several trials applied narrow inclusion criteria [12–17, 19, 20] or focused exclusively on individual lifestyle habits [12, 16, 18]. Others lacked educational components for patients and healthcare professionals regarding the connection between psychotic disorders, lifestyle, and cardiometabolic health [12–20] or failed to address systemic underdiagnosis and undertreatment of cardiometabolic conditions [12–18, 20]. Only two studies [16, 19] reported unusually low patient-to-staff ratios, while most studies [12–15, 17, 18, 20] did not report caseloads at all—an omission that limits the assessment of feasibility and generalizability to real-world psychiatric services, where higher caseloads are the norm. In addition, some interventions rely on resource-intensive strategies that are not feasible in psychiatric settings—such as providing meals [12], conducting home visits to evaluate cooking and grocery shopping, or delivering in-home physical activity coaching [19]. These factors collectively limit the scalability and long-term impact of such interventions in real-world practice.

In response to the limitations observed in previous interventions and routine psychiatric care, the LAGOM trial (Longitudinal Approach to Generate positive cardiometabolic health Outcomes in severe Mental illness) was developed as a feasible, scalable, and fully integrated intervention within psychiatric care. Conducted at the Department of Psychotic Disorders in the Greater Gothenburg region, LAGOM offers structured education to patients, their relatives, and healthcare professionals on the interplay between psychotic disorders, cardiometabolic health, and lifestyle factors. The trial adopts a holistic, person-centered approach with broad inclusion criteria, focusing on overall cardiometabolic risk rather than isolated behaviors. It emphasizes gradual, sustainable lifestyle improvements and spans a 36-month period. LAGOM also addresses underdiagnosis and undertreatment of cardiometabolic conditions through strengthened communication with primary care providers. Despite these strengths, evidence on the effectiveness of long-term, real-world interventions such as LAGOM remains limited. This trial aims to fill that critical knowledge gap.

To report this trial in a systematic and transparent way, we used the SPIRIT (Standard Protocol Items: Recommendations for Interventional Trials) 2025 [21].

## Objectives

The overall aim of the trial is to evaluate the effectiveness of the intervention in improving cardiometabolic health, promoting healthy lifestyles, and enhancing quality of life in individuals with psychotic disorders.

### Primary outcome

The primary outcome is whether the intervention is superior to usual care in reducing the estimated absolute ten-year cardiovascular risk, assessed at baseline and at 12, 24, and 36 months. Cardiovascular risk will be assessed using the Systematic COronary Risk Evaluation 2 (SCORE2) risk prediction model [22], which is recommended in European cardiovascular prevention guidelines issued by the European Society of Cardiology [23].

SCORE2 is a composite cardiovascular risk prediction model used in primary care to estimate an individual’s absolute ten-year risk of cardiovascular events. The model integrates information on several conventional cardiovascular risk factors, including age, sex, smoking status, systolic blood pressure, and non-HDL cholesterol. The outcome predicted by the model is the probability of a composite of cardiovascular mortality, non-fatal myocardial infarction, and non-fatal stroke. The CVD mortality component includes death due to coronary heart disease, heart failure, stroke, and sudden death.

#### Primary endpoint

The difference between the intervention and control groups in the mean change in estimated absolute cardiovascular risk, as assessed by SCORE2.

### Secondary outcomes

Secondary outcomes will be assessed at baseline and at 12, 24, and 36 months to:

1. Assess whether the intervention is superior to usual care in reducing cardiometabolic risk indicators.
2. Assess whether the intervention is superior to usual care in reducing the risk of CVD.
3. Assess whether the intervention is superior to usual care in reducing the risk of type 2 diabetes mellitus.
4. Assess whether the intervention is superior to usual care in improving health-related quality of life.
5. Assess whether the intervention is superior to usual care in reducing the levels of high-sensitivity C-reactive protein (hs-CRP) and HbA1c.
6. Assess cost per participant and cost-effectiveness, where cost neutrality is considered a positive outcome.
7. Assess whether the intervention is superior to usual care in improving targeted lifestyle behaviors (tobacco smoking, alcohol consumption, physical activity, and dietary habits).

#### Secondary endpoints

1. Difference in the mean change in the following cardiometabolic risk indicators:
  Body mass index (BMI) (kg/m^2^)
  Waist‒hip ratio (WHR)
  Systolic blood pressure (SBP) (mm Hg)
  Diastolic blood pressure (DBP) (mm Hg)
  Blood samples
    Plasma glucose (mmol/L)
    Total cholesterol/high density lipoprotein-cholesterol ratio (TChol/HDL-C ratio)
    Triacylglycerol/HDL-C ratio (TAG/HDL-C ratio)

2. CVD outcomes: Hazard ratio of incident CVD events

3. Diabetes mellitus outcomes: Hazard ratio of incident type 2 diabetes mellitus events
4. Biomarkers
  Difference in the mean change in hs-CRP (mg/L) and HbA1c (mmol/mol)

5. Questionnaires

- Difference in the mean change in the EQ-5D-5 L score (quality of life) [24]
- Difference in the mean change in alcohol consumption (Alcohol Use Disorders Identification Test – Consumption (AUDIT-C), scale 0–12) [25]
- Difference in the mean change in tobacco smoking per week
- Difference in the mean change in dietary habits (dietary index) (scale 0–12) [26]
- Difference in the mean change in physical activity (number of minutes per day)

6. Health economic evaluation:
  Descriptive cost analysis (average cost per participant) in SEK and EUR
  Difference in the mean change in quality-adjusted life years (QALYs) between the intervention and control groups
  Incremental cost-effectiveness ratio (ICER) based on the number of CVD or type 2 diabetes mellitus cases averted and the number of QALYs gained

### Exploratory outcomes

Exploratory outcomes will be assessed at baseline and at 12, 24, and 36 months within the intervention group to examine factors related to engagement with the intervention:

1. To assess how the number, type, and average interval between lifestyle-focused intervention sessions are associated with changes in lifestyle outcomes.
2. To assess whether participation in educational sessions (0–3 sessions) by participants and their relatives is associated with changes in lifestyle outcomes.

## Trial design

This is a longitudinal, multicenter, naturalistic, multicomponent, parallel-group, quasi-experimental cluster-based trial with a superiority framework. The trial uses a case‒control clinical design with a 1:3 allocation ratio, assigning one participant at the intervention clinics for every three at the control clinics. Clusters are defined at the level of outpatient clinics, with two intervention clinics and four control clinics.

## Methods

### Eligibility criteria

#### Inclusion criteria

1. Adults ≥ 18 years of age meeting the International Classification of Diseases, Tenth Revision (ICD-10) diagnostic criteria for any one of the schizophrenia spectrum disorders (F20-F25 or F28-F29).
2. Ability to provide informed consent.

#### Exclusion criteria

1. Having an electrical medical implant such as a pacemaker or other mechanical implants.
2. Pregnancy.
3. Deemed unsuitable by the investigator: A person may be deemed unsuitable for participation if circumstances prevent safe or reliable participation in the trial. Examples include inability or unwillingness to maintain contact with the clinic (e.g., absence of a stable address or telephone number), planned relocation or transfer to another treatment facility, municipality, or country during the trial period, mobility limitations or other practical barriers preventing attendance at clinic visits, or administrative restrictions preventing appropriate documentation in medical records or trial databases (e.g., protected or anonymous identity status). Such decisions are made on an individual basis in consultation with the clinical team (case manager (CM), treating psychiatrist, and site principal investigator (PI)) to ensure patient safety and trial integrity.
4. Prior participation in the LAGOM trial during a previous inclusion cycle (i.e., participants can only be included once during the trial period).
5. Currently under compulsory care.

## Trial setting and participant recruitment

The trial is being conducted at six outpatient psychosis clinics affiliated with Sahlgrenska University Hospital, a multi-site university hospital comprising several geographically separate hospital locations in the Greater Gothenburg region. The hospital system hosts Sweden’s largest department specializing in psychotic disorders and delivers both secondary and tertiary psychiatric care. The clinics serve individuals with psychotic disorders residing in the municipalities of Gothenburg, Mölndal, Partille, Härryda, and Öckerö, representing a total catchment area of 774,247 inhabitants (Statistics Sweden, December 31, 2024) [27].

Two clinics—Centrum (PC) and Mölndal (PM)—were purposefully selected as intervention sites due to structural and implementation-related constraints. The intervention was developed and is led by the trial leader, who is a psychiatrist based at the PC clinic. In addition, a key component of the intervention—planned bimonthly follow-up visits—is delivered by a dedicated health promoter who works across both PC and PM and is responsible for implementing core elements of the intervention.

Assigning either of these clinics to the control condition would not have been feasible, as it would have risked unintentional implementation of intervention components within usual care and introduced a high risk of contamination between trial arms. Although all participating clinics have access to health promoters as part of routine care, those working at the control clinics are not involved in the intervention and have not received training in its procedures.

Four clinics—Hisingen (PH), Nordost (PNO), Väster (PVV), and Öster (PMÖ)—serve as control sites. The allocation of clinics was therefore determined a priori to preserve implementation fidelity and minimize contamination, rather than to optimize experimental control.

A 1:3 recruitment ratio (intervention: control) was pre-specified to reflect the distribution of the underlying patient populations across participating sites. The two intervention clinics serve approximately 650 individuals with psychotic disorders, whereas the four control clinics together serve approximately 2,000 individuals. Although unequal allocation reduces statistical efficiency when total sample size is fixed, the overall sample size was increased to maintain the desired statistical power. The recruitment ratio was not determined by staffing or resource constraints, as all participating clinics operate within the same organizational structure and have comparable resources and staffing. To account for potential site-level differences, all analyses will adjust for clinics’ fixed effects.

Each participant is enrolled in the trial for approximately 36 months, with a permissible variation of ± 2 months for each annual follow-up (at 12, 24, and 36 months) to accommodate scheduling within routine clinical practice (Table 1). Consequently, the total follow-up duration may vary between 30 and 42 months. Eligible patients are identified and screened prior to their scheduled annual physical health checks. A trained representative of the clinical investigation team invites eligible patients to participate during these routine visits.

The clinical investigation team comprises healthcare professionals employed at each participating psychosis outpatient clinic, including registered and assistant nurses, occupational therapists, social workers, psychologists, and mental health support workers. These professionals also serve as CMs, who are responsible for coordinating patients’ overall care across primary care, community services, and other secondary care services, following the resource group assertive community treatment (R-ACT) model [28, 29]. The CM-to-patient ratio is approximately 1:34. In addition to the CM, the clinical investigation team includes either a psychiatrist or another attending physician (collectively referred to as “the physician”), as well as the health promoter working at the intervention clinics.

All standard CMs and physicians are involved in the trial’s implementation. Clinics may also host medical trainees (e.g., undergraduate medical students, recent graduates, or residents); however, these individuals will only be actively involved in the trial at the control clinics and will not participate in delivering the intervention.

The clinical investigation team provides full oral and written information—via a participant information sheet (PIS)—detailing the trial’s purpose, procedures, eligibility criteria, and potential risks and benefits. Participation is voluntary. Patients are informed that they may withdraw at any time without providing a reason and without consequences for their ongoing care.

Patients are given sufficient time to review the PIS, ask questions, and consider participation. The time allocated for this process is not predefined but is determined individually based on the patient’s needs and preferences; patients may choose to provide consent during the same visit or take additional time and return for a follow-up discussion before deciding. If they agree, written informed consent is obtained and signed by both the patient and the clinical investigation team representative. A copy of the signed consent and PIS is provided to the participant, and the consent process is documented in source documents and archived with essential trial materials.

The PIS specifies that if a participant withdraws, data already collected and necessary for the trial will continue to be used, but no additional data will be gathered. If new information arises that could significantly affect a participant’s health or care, it will be communicated in writing to the participant and addressed within the clinical care team. As participants receive ongoing care at the outpatient clinics, such information will be documented in the medical record and managed by the responsible healthcare professionals, with appropriate follow-up and coordination of care, including contact with primary care services when relevant. Participants may then choose whether to continue in the trial.

Interpreter-assisted consultations are routinely used at outpatient psychosis clinics and will be employed as needed during the consent process. Since the PIS is available only in Swedish, the following steps are taken to ensure comprehension:

1. Interpreter role: An accredited interpreter provides an oral translation of all trial information into the participant’s preferred language.
2. Adapted delivery: Information is presented in short sections with pauses for questions and clarification, without using technical jargon.
3. Comprehension check: Patients are asked open-ended questions to confirm their understanding (e.g., “Can you explain what the study is about in your own words?”).
4. Support inclusion: If applicable, care staff, such as housing support workers, may attend the session to reinforce understanding.
5. Decision time: Patients are given time to consider participation and may schedule a follow-up discussion before deciding.

Patients who, despite these measures, are unable to demonstrate understanding will be considered ineligible, as they do not meet the inclusion criterion: “Able to provide informed consent”. Rescreening is permitted if a patient meets the exclusion criteria at one annual check but not at the next. All clinical investigation team members involved in obtaining informed consent are trained in standardized procedures for delivering information and obtaining consent, with PIS versions tailored to intervention and control clinics. Training is coordinated by the site PIs, who ensure that only appropriately trained staff are delegated to perform consent procedures, as described in the Roles and Responsibilities section. Training activities are documented in delegation and training logs and updated as staff changes occur.

Recruitment is aligned with the routine scheduling of annual physical health checks, which are organized according to patients’ birth months. Eligible patients are therefore identified and invited to participate throughout the year as they attend their scheduled annual visits, ensuring continuous recruitment and year-round coverage. Although the planned recruitment period is 12 months, extensions may be needed due to scheduling constraints. Recruitment will continue until the target sample size is reached in both the intervention and control clinics.

A total of 650 participants will be recruited:

- Intervention clinics (PC and PM): Targeting 165 participants from a combined patient base of over 650.
- Control clinics (PH, PNO, PVV, and PMÖ): Targeting 485 participants from a combined patient base of over 2,000.

## Data collection methods

### Plans for assessment and collection of outcomes

To standardize data collection across intervention and control clinics, a structured medical history protocol—referred to as the worksheet—was developed prior to the trial. The worksheet systematized the information gathered during patients’ annual physical health checks and directly supported the research questions guiding the trial. Previously, data collection varied depending on individual healthcare professionals’ experience levels.

All healthcare professionals at the participating clinics received training in using the worksheet to interview patients approximately one year before trial initiation.

During annual physical health checks, the CM conducts a structured interview using the worksheet. While data are collected primarily through face‒to‒face interviews, additional sources include remote interviews, medical records, and self-administered questionnaires completed at home. The choice of data collection method depends on the nature and type of information being gathered. Data are collected by members of the clinical investigation team as part of routine clinical care. At the intervention clinics, these staff are also involved in delivering the intervention.

The worksheet has two versions—an intervention version (v1.1, 2025-02-21) and a control version (v1.1, 2025-01-16)—and is organized into seven data categories (Additional file 1):

1. Social and background information.
2. Medical history.
3. Lifestyle habits.
4. Results of the physical examination.
5. Blood test results.
6. Assessment scales.
7. Other information.

To support cost-effectiveness analyses, the worksheet and other trial records also capture detailed data on healthcare consumption and socioeconomic factors. These include psychiatric inpatient care (including compulsory admissions); housing status; employment; extent and duration of sick leave; permanent disability benefits; use of municipal and other support services; and dental care needs and subsidies.

For the intervention group only, data are also collected on sessions with internal or external healthcare professionals related to lifestyle behaviors, as well as on emergency room visits and hospital admissions for somatic healthcare.

Cost data are derived from the clinic’s standardized cost calculations, and the average cost of municipal services is based on pricing data from the municipalities of the Greater Gothenburg region. All costs will be inflation-adjusted over the 36-month trial period. QALYs are derived from the EQ-5D-5 L forms. This is measured using the EuroQol-5D-5 L scale [24], which is completed by the patient either at home or during annual physical health checks and is included in the worksheet. The EuroQol-5D-5 L scale has been validated in people with schizophrenia [30].

Additionally, data on causes of death are available through clinic and trial records, with access to relevant medical records obtained as part of the informed consent process.

#### Measurements

1. Blood pressure and pulse:
  Blood pressure and pulse were measured on the right arm after 15 min of seated rest using the OMRON HEM-907-E7. The device is set to *AVERAGE* mode, which calculates the mean of two readings taken 60 s apart. The *P-SET* is set to *AUTO* to automatically adjust the cuff pressure. Cuffs are selected based on arm size and are available in three sizes (medium, large, and extra-large; 22–50 cm range). All CMs are trained in proper device use.

2. Weight:
  Weight was measured using the SECA 799 scale with participants wearing indoor clothing and no shoes. The values are rounded to the nearest whole number.

3. Body composition:
  Body fat (%), bone mass (kg), body water (%), muscle mass (kg), and metabolic age are measured using the TANITA DC-430MA. The participants are barefoot and wear indoor clothing; 1 kg is subtracted to account for clothing.

4. Height:
  Measured wearing socks, without shoes, on a firm, flat surface using a calibrated, wall-mounted stadiometer, with the head in the horizontal plane. Rounded to the nearest whole number.

5. Waist and Hip Circumference:
  Measured using a flexible, non-stretch, multi-color, dual-sided anthropometric measuring tape (150 cm/59 in).
  Waist circumference is measured at the level of the umbilicus during exhalation. This approach was selected to improve feasibility, patient comfort, and measurement consistency in routine clinical practice, particularly in individuals with higher body mass, where standard anatomical landmarks may be difficult to identify reliably. Different measurement protocols have been proposed in the literature, and no single method is universally adopted; therefore, consistency within the study was prioritized [31, 32]. Hip circumference is measured at the widest part over the buttocks. All values are rounded to the nearest whole number.

6. Timing of Measurements:
  The time of each physical exam is recorded to allow for time-adjusted analyses.

All CMs and physicians involved in the trial received standardized training to perform these measurements consistently. Ongoing training and oversight are ensured by the site PIs, as described in the Roles and Responsibilities section.

#### Blood tests

Blood tests are ordered by the physician via CM as part of routine clinical procedures. Patients receive both oral and written instructions for preparing for the blood tests. These include the following standard recommendations: patients must fast for at least 10 h before the test. If they choose to drink coffee or tea during the fasting period, they are instructed not to add sugar or milk. Morning medications should be taken only after the blood sample has been collected. Patients are also advised that alcohol consumption and intense physical activity the day before testing may influence the test results.

#### QRISK3

QRISK3 (version 2018.0) is a validated tool for estimating 10-year CVD risk, accounting for psychotic disorders and antipsychotic use as independent risk factors [33].

In this trial, the QRISK3 is included only in the intervention worksheet as a visual, motivational aid, illustrating how changes in smoking, weight, blood pressure, and lipids can lower overall risk. It also demonstrates the synergistic effect of multiple small improvements, fostering a shared understanding between participants and healthcare professionals. The QRISK3 is used solely for lifestyle counseling and does not influence clinical decision-making.

#### Body composition analyzer

The TANITA DC-430MA is a CE-marked class IIa medical device that uses dual-frequency bioelectrical impedance analysis to measure weight, BMI, body fat, muscle mass, visceral fat, basal metabolic rate, and other composition metrics. It is widely used in medical and research settings for screening and monitoring lifestyle-related conditions. In this trial, it is employed to support lifestyle counseling, in line with its intended purpose, without influencing medical decisions. The results are shared only with participants.

#### Training of the clinical investigation team

All the clinical investigation team members attended a one-day workshop on standardized measurements of blood pressure, waist circumference, and height. The staff at the intervention clinics received additional training in the use of the QRISK3 and the TANITA body composition analyzer.

### Plans to promote participant retention and complete follow‑up

A patient-centered approach is embedded throughout all trial procedures and interactions. The CM at each outpatient clinic serves as the primary point of contact for participants and is available to address questions, offer support, and respond to any concerns that may arise during the trial.

To minimize participant burden, trial visits—including annual health checks and bimonthly physical examinations—are scheduled, when possible, to coincide with existing clinic or outpatient appointments. Since annual health checks are already part of routine care, integrating research activities into these visits helps reduce additional time commitments.

In addition, annual education sessions and bimonthly physical examinations are integral components of the patient-centered approach and are expected to further strengthen participant engagement and retention by fostering ongoing interaction, motivation, and continuity of care.

Participants receive travel reimbursements for attending bimonthly physical examinations and annual education sessions. CMs are responsible for maintaining ongoing contact with participants and coordinating the scheduling of all trial-related visits. CMs and research assistants, who are medical secretaries and administrators at the outpatient clinics, support retention efforts by tracking attendance and assisting with logistical tasks such as sending reminders and coordinating visits to align with participants’ routines. These activities reflect routine administrative and coordination tasks within outpatient clinic workflows.

This proactive, coordinated approach is intended to support participant engagement and promote long-term retention over the 36-month follow-up period.

## Confidentiality, data management, and access to data

All trial data will be registered, managed, and stored to ensure accurate reporting, interpretation, and verification while maintaining participant confidentiality. This is detailed in a Data Management Plan (DMP) (version 1.0, 2025-02-05) approved by the trial sponsor.

At each site, the PI—who also serves as the research nurse—will collect completed worksheets from the CMs and physicians and store them in physical folders organized by participant. Data are gathered using paper-based methods. Within 45 days of the second visit of the annual physical health check, the PI will verify that each worksheet is complete and accurate. Any missing or questionable data will be clarified using information from the participant, CM, physician, or medical records. Once validated, the worksheet will be transferred to a research assistant for data entry into the electronic case report form (eCRF).

Data entry into the eCRF will also be completed within 45 days of the second visit. During entry, the research assistant identifies missing or abnormal values, such as swapping weight and height values, reversed blood pressure readings, or inconsistencies (e.g., no somatic illness reported despite the use of antihypertensive or antidiabetic medication). Any such discrepancies are communicated to the responsible CM and physician for clarification and correction, with the aim of preventing similar issues in the future.

The eCRF system is managed using Research Electronic Data Capture (REDCap), which serves as the electronic data capture (EDC) tool for this trial. The current version in use is v.14 (dated 30 November 2023), supplied by Gothia Forum and hosted by Region Västra Götaland (VGR-IT). REDCap provides a secure interface for data entry, supports real-time data validation, maintains complete audit trails for tracking data modifications, and enables data export to common statistical packages. It also allows data import from external sources. All data are stored on encrypted servers protected by firewalls, with daily backups and individual user logins secured by two-factor authentication. Access to the REDCap system is restricted to trained and authorized personnel, with role-based permissions (e.g., data editing or read-only access).

Once the data have been finalized, a formal “Clean File” decision is documented in a ‘Clean File form’ document. At this point, access to the eCRF will be withdrawn, except for authorized data export. PIs at each site retain access to cleaned datasets from their respective sites and may request access to data from other sites.

To ensure confidentiality, each participant is assigned a unique record ID, which is used for all data collection, storage, and analysis. A participant enrollment and identification list is maintained separately, linking each participant’s name and personal identification number to that participant’s record ID. The eCRF contains only data linked to a unique record ID and does not include personal identification numbers, thereby minimizing the risk of confidentiality breaches. Paper records and forms containing personal information are stored securely in locked areas at each outpatient clinic and are accessible only to authorized staff. These materials will never be left in public or unsecured spaces. Additionally, results will only be reported at the group level, ensuring that no individual participant can be identified. As part of the trial documentation, an entry is made in each participant’s medical record documenting their enrollment in the trial and what it entails.

The informed consent process complies with applicable data protection and privacy legislation. Participants are fully informed about how their data will be collected, used, and published, and how confidentiality will be protected. The PIS also states that authorized representatives of the sponsor or regulatory authorities may access relevant medical or trial records—including the participant’s medical history—for purposes of data verification.

## Archiving

The sponsor representative and PI will maintain the essential trial documents in the Trial Master File (TMF) and Investigator Site File (ISF), respectively. Both the sponsor representative and PI will archive their respective documentation and data for at least 10 years after the trial ends, in accordance with the Medical Device Regulation (MDR, Annex XV) and institutional information management policies. Access to trial data is governed by clinical trial agreements between the sponsor and participating sites.

## Quality assurance and quality control

Quality assurance ensures high-quality data collection, whereas quality control detects and addresses data problems promptly.

Our quality assurance approach involves (1) creating a flowchart for the intervention; (2) developing intervention and control worksheets; (3) conducting workshops to train data collectors; (4) holding regular meetings with data collectors; (5) maintaining logs of training sessions; and (6) following the manufacturer’s maintenance instructions for the OMRON blood pressure devices, SECA scales, and TANITA body composition analyzers.

Our quality control strategy includes (1) monitoring the completed worksheets via research nurses and research assistants; (2) rectifying missing or incorrect worksheet data through research nurses during the first review and through research assistants prior to database entry; (3) tracking data entry delays; and (4) conducting regular meetings with research nurses and assistants to address missing, out-of-range, or illogical data.

## Harms

Safety monitoring, adverse event definitions, reporting procedures, and causality assessment for this clinical trial follow applicable regulatory guidelines. Causality is assessed using a standardized 4-level scale, and all reporting procedures follow applicable regulatory timelines. Full details are described in the Clinical Investigation Plan (CIP), which is publicly available at ClinicalTrials.gov (NCT06781801). The CIP is the formal protocol describing the objectives, design, methodology, monitoring, statistical considerations, and organization of the clinical investigation, as defined in SS-EN ISO 14155:2020. In some documents, this may be referred to simply as the Protocol.

## Intervention and comparator

### Control clinics (usual care)

Usual care refers to the existing clinical practices for individuals with psychotic disorders in the Greater Gothenburg region. In Sweden, all citizens are registered with a primary care center, which is responsible for managing cardiometabolic risk factors—even for patients receiving ongoing specialist psychiatric care.

In the Greater Gothenburg region, individuals with psychotic disorders receive annual physical health checks at psychosis outpatient clinics as part of usual care. This process typically includes two visits.

### Annual physical health checks

#### Baseline Visits

##### Visit 1 – The annual physical health check with CM

As part of usual care, the CM prepares for the annual physical health check by ordering blood tests, beginning to fill in the worksheet, and sending a letter to the patient with instructions for blood sample preparation. Depending on the case, the CM may also send self-assessment questionnaires to be completed at home (including EQ-5D-5 L, AUDIT-C, and a questionnaire on dietary habits). These procedures are part of routine care and are unaffected by participation in the trial.

During the visit, the CM completes the control version of the worksheet, performs the physical examination, and ensures that fasting blood tests are conducted. Visit 1 lasts approximately 60 min. The blood tests and physical examination provide the cardiometabolic parameters relevant to this trial, as outlined in Table 1.

##### Visit 2 – The annual physical health check with the physician and CM

The physician and CM review the worksheet, laboratory, and physical examination results. Visit 2 lasts approximately 60 min. Clinical management is guided by the physician’s judgment, which is typically based on reference values for cardiometabolic parameters. The participant, CM, and physician collaborate to determine a suitable management plan based on outpatient clinic’s routines. Usual care may include basic lifestyle advice or referrals to health promoters, dietitians, or primary care professionals.

The trial does not modify the delivery of usual care but aims to have the two annual visits for each participant occur within a 45-day period to support consistency in data collection. This timeframe is not a strict requirement, and participants are not excluded if visits occur outside this window.

However, it may not always be feasible for patients to undergo blood tests or physical examinations on the scheduled days of these visits. To accommodate this, the protocol allows these assessments to be completed up to 45 days after Visit 2. This establishes a visit window of **±** 45 days from the second visit. If either the blood tests or physical examinations are completed after Visit 2, a follow-up physician appointment is scheduled to review the results and make appropriate clinical decisions. This visit window is designed to reflect the realities of clinical practice while maintaining consistency in data collection.

#### Continuing annual physical health checks

Follow-up assessments at months 12, 24, and 36 replicate the baseline procedure.

##### Visits 3, 5, and 7 – The annual physical health check with CM

The same routine as in Visit 1 (baseline) at the control clinics (Table 1).

##### Visits 4, 6, and 8 – The annual physical health check with the physician and CM

The same routine as in Visit 2 (baseline) at the control clinics (Table 1).

Patients not enrolled in the trial will continue to receive usual care at the control clinics.

#### Intervention clinics

**Fig. 1:**
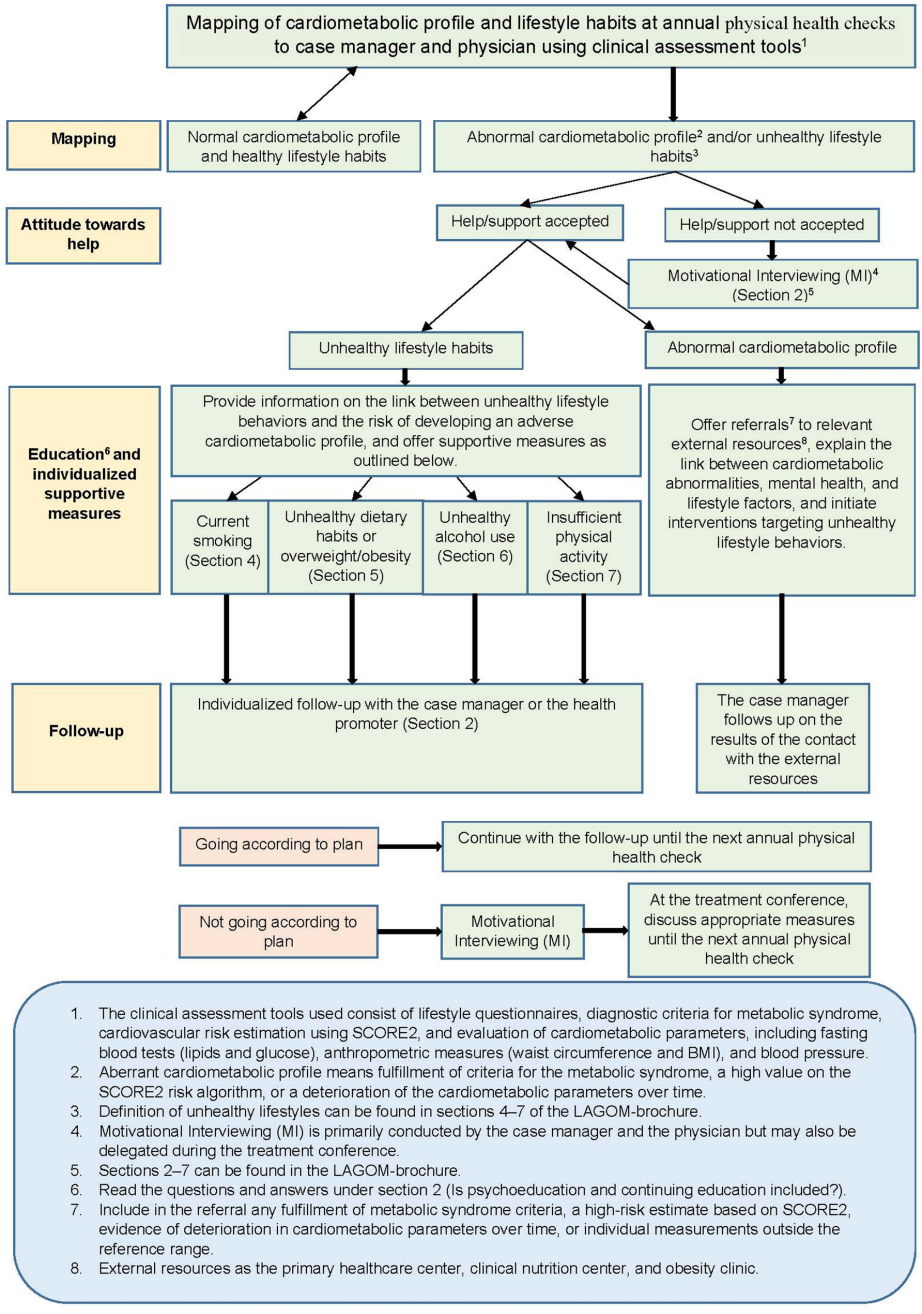
Flowchart

At the intervention clinics, participants receive enhanced assessments and individualized support integrated into usual care. The intervention follows a structured and manualized approach guided by a flowchart (Fig. 1), an intervention-specific worksheet, and a brochure (Additional file 2). The brochure describes the trial and defines the lifestyle habits adapted for individuals with psychotic disorders. It is designed to support healthcare professionals in delivering interventions consistently and effectively. Together, these tools organize the workflow and ensure systematic implementation across the healthcare professionals involved in care. The intervention is designed to promote cardiometabolic health through individualized assessment, feedback, and follow-up.

### Annual physical health checks

The annual physical health checks at intervention clinics follow the same two-visit structure as usual care but include additional assessments, motivational tools, and follow-up mechanisms tailored to address cardiometabolic health in patients with psychotic disorders.

#### Baseline visits

##### Visit 1 – The annual physical health check with CM

This visit mirrors the control clinic structure and lasts approximately 60 min but includes additional components:

- The CM uses the intervention version of the worksheet.
- Body composition is assessed using the TANITA DC-430MA analyzer, as outlined in Table 1.
- No intervention-specific procedures are initiated before written informed consent is obtained.

##### Visit 2 – The annual physical health check with the physician and CM

This visit lasts approximately 60 min and has the following additional components compared with usual care:

1. Individualized and comprehensive mapping of cardiometabolic risk and lifestyle habits beyond standard cut-off values. This includes assessment of SCORE2 and metabolic syndrome [34] criteria to provide a contextual estimate of the risk for CVD and diabetes, as well as evaluation of trends in risk factors over time.
2. Assessment of risk behavior in lifestyle habits is linked to the participant’s state of cardiometabolic health as well as to the gains the participants make by stopping their unhealthy lifestyle.
3. The physician fills in the QRISK3 algorithm with the participant.
4. The physician uses the results of visual motivational tools—QRISK3 and the TANITA—to support patient understanding and encourage engagement in lifestyle change and cardiometabolic risk management.
5. The physician, CM, and participant jointly develop a personalized care and follow-up plan.
6. Risk-oriented referrals to internal resources (e.g., health promoters, physiotherapists) for further assessment and support or to external providers (e.g., primary care) to promote appropriate diagnosis and management of cardiometabolic concerns, with an emphasis on individualized care. CMs coordinate and follow up on referrals.

### Additional intervention components

1. Coordination, motivational support, and counseling.
  The CM conducts individualized follow-up sessions (15–30 min), in person or remotely.
  Sessions target key lifestyle factors: diet, physical activity, alcohol, and tobacco use.
  The frequency, content, and delivery mode of the sessions are tailored to the participants’ needs.
  These sessions ensure engagement with care plans initiated by psychiatry or agreed-upon external healthcare resources (e.g., primary care or dietitians) and support continuity, adherence, and necessary adjustments.
  The approach emphasizes small, sustainable adjustments that consider cognitive limitations and stress sensitivity.

2. Bimonthly follow-up with physical examination.
  Every two months, participants attend a clinic visit that includes a focused discussion of relevant lifestyle behaviors and a physical examination.
  The measurements included SBP, DBP, pulse, weight, waist and hip circumference, and body composition, which are assessed using the TANITA analyzer (Table 1).
  These regular assessments are used to monitor progress and guide individualized adjustments to the care plan.

3. Group education sessions for participants and relatives.
  Once annually, participants and their relatives are invited to a 45-minute in-person education session.
  A representative from the clinical investigation team will hold the education sessions.
  Sessions are based on the LAGOM concept and focus on the connection between lifestyle, cardiometabolic health, and psychotic disorders.
  The participation of relatives is optional; no data are collected from them.

4. Education sessions for staff.
  Internal education seminars are held biannually.
  These address the interplay between cardiometabolic risk, psychotic disorders, and lifestyle habits.
  Seminars are grounded in the LAGOM framework and promote consistent, evidence-based practice among healthcare professionals.

### Ongoing data collection and adherence

The intervention includes a structured plan for monitoring implementation fidelity and patient adherence:

- The CM is responsible for documentation during annual check-ups.
- Contact with referred services is tracked.
- Follow-ups aim to reinforce intervention goals and evaluate effectiveness.
- The CM documents the number and type of sessions with internal and external resources in the worksheet at the next annual health check, based on self-reports and medical and trial records.

#### Continuing annual physical health checks

Participants undergo follow-up assessments at months 12, 24, and 36, which replicate the baseline procedures:

##### Visits 8, 15, and 22 – The annual physical health check with the CM

The same routine as in Visit 1 (baseline) at the intervention clinics (Table 1).

##### Visits 9, 16, and 23 – The annual health check with the physician and the CM

The same routine as in Visit 2 (baseline) at the intervention clinics (Table 1).

##### **Visits 3–7**,** 10–14**,** and 17–21**: Bimonthly physical examinations, as outlined in Table 1

Participants initially deemed ineligible may be rescreened. Patients not enrolled in the trial at intervention clinics continue to receive usual care.

#### Awareness of assignment

Due to the nature of the intervention, both participants and healthcare professionals are aware of the clinic assignment.

**Table 1:** Schedule of enrollment, interventions, and assessments

Collected data	Baseline		Every other month ± 14days Visits 3–7*	12 months ± 60 days Visits 8 + 9* Visits 3 + 4	Every other month ± 14 days Visits 10–14*	24 months ± 60 days Visits 15 + 16* Visits 5 + 6	Every other month ± 14 days Visits 17–21*	36 months ± 60 days Visits 22 + 23* Visits 7 + 8
	Visit 1	Visit 2^#^						
**Check for eligibility**	**X**							
**Informed consent**	**X**							
Inquire about adverse event*			**X**	**X**	**X**	**X**	**X**	**X**
**Social and background questions**	**X**			**X**		**X**		**X**
**Medical anamnesis**	**X**			**X**		**X**		**X**
Questions about participation in educational sessions and lifestyle sessions*	**X**			**X**		**X**		**X**
**Lifestyle questionnaires**								
• Alcohol habits according to AUDIT-C • Tobacco habits • Dietary habits • Physical activity	**X**			**X**		**X**		**X**
**Blood tests**								
P-high-sensitivity CRP (mg/L)	**X**			**X**		**X**		**X**
fP-Triacylglycerol (mmol/L)	**X**			**X**		**X**		**X**
P-HDL-cholesterol (mmol/L)	**X**			**X**		**X**		**X**
P-LDL-cholesterol (mmol/L)	**X**			**X**		**X**		**X**
P-Total cholesterol (mmol/L)	**X**			**X**		**X**		**X**
P-non-HDL-cholesterol (mmol/L)	**X**			**X**		**X**		**X**
fP-Glucose (mmol/L)	**X**			**X**		**X**		**X**
B-HbA1c (mmol/mol)	**X**			**X**		**X**		**X**
P-Kreatinin (µmol/L)	**X**			**X**		**X**		**X**
P-ALT (µkat/L)	**X**			**X**		**X**		**X**
P-AST (µkat/L)	**X**			**X**		**X**		**X**
P-ALP (µkat/L)	**X**			**X**		**X**		**X**
P-bilirubin (µmol/L)	**X**			**X**		**X**		**X**
**Physical examination**								
Height (cm)	**X**			**X**		**X**		**X**
Weight according to SECA 799 (kg)	**X**		**X**	**X**	**X**	**X**	**X**	**X**
Waist circumference (cm)	**X**		**X**	**X**	**X**	**X**	**X**	**X**
Hip circumference (cm)	**X**		**X**	**X**	**X**	**X**	**X**	**X**
Systolic blood pressure mmHg	**X**		**X**	**X**	**X**	**X**	**X**	**X**
Diastolic blood pressure mmHg	**X**		**X**	**X**	**X**	**X**	**X**	**X**
Pulse (bpm)	**X**		**X**	**X**	**X**	**X**	**X**	**X**
**Assessment scales**								
EQ-5D-5 L	**X**			**X**		**X**		**X**
Fulfillment of criteria of metabolic syndrome*	**X**			**X**		**X**		**X**
SCORE2*	**X**			**X**		**X**		**X**
QRISK3*		**X**		**X**		**X**		**X**
Measurements according to TANITA body composition analyzer*								
• Weight (kg) • Total body fat mass (kg) • Total body water mass (kg) • Total body muscle mass (kg) • Bone mass (kg) • Metabolic age	**X**		**X**	**X**	**X**	**X**	**X**	**X**

* Only intervention clinics

# Visit 2 complements Visit 1 by ensuring completeness of the data collected during the first visit. This allows the physician to base decisions on a full assessment. Eligibility screening is always completed during Visit 1, while informed consent may be obtained at either Visit 1 or Visit 2

### Criteria and procedures for participant withdrawal or discontinuation

Participants have the right to withdraw from the trial at any time without providing a reason and without any impact on their ongoing or future treatment.

If a participant discontinues the trial, follow-up and care will continue in accordance with the outpatient clinic’s routines.

Individual participant discontinuation may occur under the following circumstances:

- Enrollment in violation of the inclusion or exclusion criteria (inappropriate enrollment).
- Withdrawal of informed consent.
- Pregnancy.
- Receipt of an electronic medical implant (e.g., pacemaker) or other mechanical implants.
- Relocation to another city or country, or registration at a different outpatient clinic.
- Revision of diagnosis such that the participant no longer meets ICD-10 criteria for a schizophrenia spectrum disorder.

When available, reasons for participant discontinuation or withdrawal will be documented in the eCRF and reported in the trial flow chart.

### Patient engagement

Prior to the start of the trial, members of the Schizophrenia Association in Gothenburg were involved in revising the intervention flowchart and brochure, as well as developing and revising the worksheets. These members included individuals with lived experience of psychotic disorders and organizational representatives. They have also reviewed the content and structure of the educational sessions for participants and their relatives to help ensure that the sessions are relevant, understandable, and appropriately tailored to participants’ needs.

Patients and/or the public were not involved in the reporting or dissemination plans of this research.

### Sample size

The sample size was calculated to detect a clinically meaningful reduction in the estimated cardiovascular risk score derived from the SCORE2 model. A clinically meaningful effect was defined as a 20% relative reduction in estimated ten-year cardiovascular risk at follow-up, corresponding to an absolute reduction of approximately 2.2% points, assuming a baseline mean SCORE2 value of 11 [35] and a standard deviation of 5.5 [36]. A total of 66 participants in the intervention group and 198 in the control group would be required to detect this difference with 80% statistical power at a two-sided significance level of 0.05.

Because participants are recruited from multiple clinics, the sample size was adjusted for clustering. Assuming an intraclass correlation coefficient of 0.01 for patients within clinics [36] and an anticipated dropout rate of 40% over the 36-month follow-up period, the required sample size increases to 165 participants in the intervention group and 485 participants in the control group. The sample size was calculated using the statistical package STATA (StataNow/SE 19.5).

### Statistical and health economic analysis plan

The trial involves longitudinal comparisons both within and between the intervention and control groups.

#### Primary outcome analysis

Cardiovascular risk (SCORE2): Analyzed using linear mixed-effects regression models for repeated measures.

#### Secondary outcome analysis

- Cardiometabolic indicators — BMI, WHR, SBP, DBP, the TAG/HDL-C ratio, the TChol/HDL-C ratio, and plasma glucose — will be analyzed using linear mixed-effects regression models for repeated measures. Data will be drawn from assessments conducted within a ± 45-day window surrounding the physician-led annual physical health check. Sensitivity analyses will examine the impact of:
  - Including cardiometabolic data collected outside the predefined visit window.
  - Outliers, by comparing models with and without their inclusion.
  - Missing data and dropouts.
- Time-to-event outcomes (e.g., incident or recurrent cardiovascular events and incident type 2 diabetes mellitus): Analyzed using Cox proportional hazards regression and Kaplan–Meier survival estimates.
- Quality of life (EQ-5D-5 L): Analyzed using linear mixed-effects regression models for repeated measures.
- Inflammatory and metabolic markers (hs-CRP and HbA1c): Analyzed using linear mixed-effects regression models for repeated measures.
- Lifestyle behaviors (physical activity, dietary habits, alcohol consumption, and tobacco smoking): Analyzed using linear mixed-effects regression models for repeated measures.

#### Health economic analysis

The health economic evaluation will consist of three components:

1. Descriptive cost analysis.
  Direct and indirect costs will be calculated and reported in both SEK and EUR.
  Costs will be indexed to the clinical trial’s end date.

2. QALYs.
  Derived from EQ-5D-5 L scores (5-dimensional scale) and analyzed using linear mixed-effects regression models for repeated measures.

3. ICER.
  The ICER will be calculated based on cost per:
    Case of CVD averted,
    Case of type 2 diabetes mellitus averted, and.
    QALY gained based on EQ-5D-5 L scores normalized to a [0, 1] interval.

Sensitivity analyses will test the robustness of the cost-effectiveness model using a ± 20% variation in key input parameters. Trial management costs will be excluded from the analysis. However, the model will incorporate the additional time required to deliver the intervention (e.g., more frequent or longer visits), alongside the time spent on usual care. Achieving cost neutrality for both direct and indirect costs will be viewed as a positive outcome.

#### Exploratory outcome analysis

Within the intervention group, linear mixed-effects regression models for repeated measures will be used to assess associations between the number, type, and average interval between lifestyle-focused intervention sessions, as well as participation in educational sessions, and changes in lifestyle behaviors—including smoking status (number of cigarettes per week), AUDIT-C score, dietary index, and time spent in physical activity.

All primary, secondary, and exploratory outcomes will be analyzed using two-sided statistical tests with a Type I error rate (α) of 0.05. All models will be adjusted for potential confounders, including age, sex, the fixed effects of sites, the interval between consecutive follow-up visits, and any baseline covariates not balanced between groups.

### Missing data

A two-step strategy is in place to minimize the occurrence of missing data.

1. Initial Review: The PI reviews the completed worksheets after the second visit of the annual physical health check to identify any missing or inconsistent entries.
2. Follow-up Review: A research assistant then performs a secondary check. Both checks are conducted within 45 days of the second visit of the annual physical health check, unless delayed owing to limited staff availability and scheduling constraints during summer, Christmas, or national holidays.

In addition to manual review, the eCRF includes built-in validation checks for missing values and implausible entries.

To inform our handling of missing data during analysis, we will evaluate:

- Level of missing data: item-level, construct-level, and person-level;
- Missing data mechanism: whether the data are missing completely at random (MCAR), missing at random (MAR), or missing not at random (MNAR) [37].

Based on this assessment, appropriate handling strategies will be applied, such as Full Information Maximum Likelihood (FIML), Multiple Imputation (MI), or listwise/pairwise deletion. Sensitivity analyses will be performed to assess how missing data may influence results and to evaluate the robustness of the selected handling methods.

### Monitoring

The trial organization includes a steering and coordination structure consisting of the coordinating investigator, site principal investigators, research assistants, and the health promoter, who together are responsible for the integrity, conduct, and progress of the trial. Oversight is further supported by two members of the Research, Development, Education and Innovation Council at the Department of Psychotic Disorders and by an independent statistician affiliated with another university. In addition, the trial will undergo external risk-based monitoring conducted by an independent monitor from the Scandinavian Clinical Research Organisation (SCRO), which is appointed by the sponsor. Monitoring will take place before the trial begins, during its conduct, and after its completion to ensure compliance with the CIP, Good Clinical Practice (GCP), SS-EN ISO 14155:2020, and applicable ethical and regulatory requirements, including the Declaration of Helsinki.

Given the low-risk nature of the intervention and the absence of anticipated safety concerns, no formal Data Monitoring Committee was established. External independent oversight is provided through risk-based monitoring conducted by the SCRO, whose monitor is independent of the sponsor representative, PI, and trial site staff.

### Purpose and approach

According to International Council for Harmonisation of Technical Requirements for Pharmaceuticals for Human Use–Good Clinical Practice (ICH-GCP) and applicable regulations, the sponsor is responsible for ensuring the trial is quality-controlled through monitoring. The monitor must have access to all trial materials, and site staff must allocate time for monitoring activities. Monitoring is risk-based and guided by a Monitoring Plan (version 1.0, dated 24 February 2025), approved by the trial sponsor, developed before the start of the trial, and subject to adjustment based on changes to the trial or updated risk assessments.

The sponsor has assessed this trial as low-risk, allowing for a reduced intensity of monitoring. This classification is based on the absence of known risks with the investigational devices (QRISK3 algorithm and body composition analyzer) and the fact that the trial is embedded in routine clinical practice, where risks are no greater than usual care. The main potential burden is the possibility of additional visits for the intervention group; to mitigate this, participants are exempt from visit costs and receive transportation support.

Given the potential clinical benefits—improved detection and management of cardiometabolic risk factors, enhanced quality of life, and cost-effective care for individuals with psychotic disorders—the expected benefits clearly outweigh the minimal risks. Continuous internal quality control will be conducted by the sponsor’s research team to ensure adherence to the CIP and data integrity.

Monitoring ensures:

- The rights, safety, and well-being of trial participants are protected.
- Data collected are accurate, complete, and consistent with source documents.
- The trial was conducted in accordance with the approved protocol, GCP, and applicable ethical and regulatory requirements, such as the Declaration of Helsinki.

### Participant confidentiality

As described in the informed consent form, participants explicitly consent to allow authorized representatives of the sponsor, including the monitor and relevant regulatory authorities, access to relevant parts of their medical and trial records—including medical history—for purposes of data verification. This is communicated clearly during recruitment through both oral and written information. Access will also be granted for regulatory inspections.

### Monitoring visits

SCRO’s monitor will conduct the following types of monitoring visits across the six sites in the Greater Gothenburg region:

1. Study initiation visit:
  Verifies staff training and site readiness prior to participant enrollment.
  Confirms the availability of all essential documents and procedures.
  Ensures that the ISF is complete.
  Four initiation visits are conducted: one for the two intervention sites and three for the four control sites.

2. Monitoring and Source Data Verification.
  Conducted at four to six planned visits per site during the trial period.
  Includes review of informed consent forms, source data, inclusion/exclusion criteria, QRISK3 and TANITA results, and physical examination variables (e.g., weight, blood pressure).
  20% of records at each site will be reviewed, with 100% verification for all serious adverse events (SAEs) in the intervention group.
  Ensures that the eCRF is completed correctly and queries are issued as needed.
  A source data verification agreement is signed prior to participant inclusion at each site.

3. Site closure visits:
  Occurs after the last participant completes the trial and a Clean File has been declared.
  The sponsor representative will confirm when a site is ready for closure.
  Verifies final documentation (e.g., Clean File documents, signed logs, informed consents).

4. Remote Monitoring:
  Includes review of eCRF data entry and query management after the first 20 participants at each site complete baseline visits.

All monitoring activities will be conducted according to the monitoring plan developed by the sponsor, which includes a predefined schedule and standard procedures.

### Documentation and follow-up

After each visit, the monitor will issue a monitoring report within 10 working days. The report will detail monitoring findings, proposed actions, and responsible parties. It is signed by both the monitor and the sponsor representative and stored in the TMF and ISF. Signed originals are maintained by the sponsor representative and will be archived after trial closure.

### TMF and ISF management and oversight

Gothia Forum at Sahlgrenska University Hospital supported the preparation of both the TMF and ISF in collaboration with the sponsor representative. Ongoing management of the TMF will be coordinated with the sponsor representative, while each trial site is responsible for maintaining its own ISF. Essential documents will be maintained throughout the trial, with appropriate archiving procedures implemented at its conclusion. The SCRO monitor will oversee the completeness, accuracy, and regulatory compliance of both files in accordance with the monitoring plan.

### Source data and access to documentation

The PI at each site is responsible for maintaining source documents for every participant throughout the trial. Prior to trial initiation at each individual site, the definition and location of source data were determined in collaboration with the monitor and documented in the **Source Data Location Agreement**, which is included in the ISF. This agreement specifies what constitutes source data at each site (e.g., medical records, worksheets).

In cases where certain data are not documented elsewhere, the worksheet has been designated as the source document, as agreed upon with the monitor and documented in the Source Data Location Agreement.

Access to all trial-related documentation—including medical records, worksheets, eCRFs, and other trial documentation—will be provided for monitoring and other quality control activities.

### CIP version

Version: Version 3.0/Amendment 1 (dated 10 June 2025).

Replaces: Version 2.0 (dated 6 December 2024).

#### Revision

The substantial modification involves a change of PI at two outpatient clinics (PNO and PMÖ). In addition, a clarification has been added specifying that both Visit 1 and Visit 2 are part of the annual physical health check, with both visits occurring within a 45-day window. Each visit will last approximately 60 min. Additional minor, nonsubstantial changes have also been made to the CIP and the PISs.

#### Rationale

The PI changes are necessary due to leadership updates at the respective clinics. The clarification regarding visit structure and timing was made to ensure consistency and transparency across all participating sites. Minor adjustments to the CIP and PIS were implemented to improve clarity and alignment with current trial procedures.

Version: Version 3.1 (dated 21 October 2025).

Replaces: Version 3.0 (dated 10 June 2025).

#### Revision

This version includes minor, nonsubstantial updates to improve clarity, consistency, and completeness:

- “Care as usual” changed to “usual care” throughout the document.
- “Intervention group” and “control group” revised to “intervention clinics” and “control clinics,” respectively, where applicable.
- Table 1: “Eating habits” changed to “Dietary habits.”
- Table 1: Timing of data collection for “Questions about participation in educational sessions and lifestyle sessions” specified at baseline, 12 months, 24 months, and 36 months.
- Sect.  6.3.2 (Exclusion Criteria): Clarified wording for “Deemed unsuitable for inclusion at the discretion of the investigator” and “Previous participation in the clinical trial.”

#### Rationale

These updates were made to enhance clarity, ensure internal consistency, and provide complete documentation of data collection and eligibility procedures. All changes are minor and nonsubstantial, with no impact on the trial design, methodology, or participant safety.

### Protocol amendments

Any amendments to the CIP are developed and agreed upon by the sponsor in consultation with the coordinating investigator. Substantial amendments—defined as changes that may impact participant safety, the scientific value of the trial, or the conduct of the trial—must be submitted to and approved by the Swedish Ethical Review Authority and/or the Swedish Medical Products Agency before implementation. Nonsubstantial amendments that do not affect participant safety or trial integrity are documented in writing, dated, and filed in both the TMF and the ISF, with notification to relevant parties as required.

All amendments are tracked through version control and documented in the Version history described in the Ethics approval, consent, and compliance section, ensuring full traceability of changes and their rationale. Updated versions of the CIP and related trial materials are distributed to all participating sites, and site staff are trained on the changes before implementation.

### Deviations from the protocol

Investigators may not deviate from the protocol except to protect a participant’s rights, safety, or well-being in emergency circumstances.

All such deviations must be documented with an explanation and reported to the sponsor representative as soon as possible. The sponsor representative will review the deviations and, when applicable, report them to the Swedish Medical Products Agency and/or the Swedish Ethical Review Authority.

### Insurance

The participants in the trial will be covered by Swedish Patient Insurance and liability insurance.

### End of the trial

The trial will conclude when the last participant has completed the final scheduled visit (Visit 8 at the control clinics and Visit 23 at the intervention clinics; see Table 1 for the visit schedule). The sponsor representative will notify the Swedish Medical Products Agency within 15 days after the end of the trial and send the trial report within 1 year after the end of the trial, including an easily understandable summary.

### Trial organization

The trial is being conducted at six psychosis outpatient clinics (sites) in the Greater Gothenburg region. Each site has a PI and research assistant. The two intervention sites also share a health promoter. Together, these staff form the core research team, which is responsible for the integrity, coordination, and progress of the trial. Oversight is further supported by two members of the Research, Development, Education and Innovation Council at the Department of Psychotic Disorders and by an independent statistician affiliated with another university.

#### Roles and responsibilities

- The main author of this article (HN), a specialist in psychiatry, represents the sponsor, Region Västra Götaland, and serves as the coordinating investigator leading the trial. HN oversees implementation, conducts interviews, performs data analysis and manuscript preparation, and holds regular site meetings.
- The PIs, specialist nurses in psychiatric care, coordinate site activities, maintain the ISF, and train CMs on trial procedures (e.g., eligibility screening, consent, physical exams). They also conduct interviews and manage delegation logs, training records, participant scheduling, and subject enrollment and identification logs. At intervention sites, PIs provide additional training on QRISK3, TANITA, and other intervention-specific routines. Duties may be delegated to co-PIs as needed.
- Research assistants support data entry, error checking, eligibility screening, attendance tracking, trial material management, and sending out health check invitations. At intervention sites, they also handle transport logistics.
- The health promoter (intervention sites) coordinates bimonthly follow-up visits, which include physical examinations, patient scheduling, attendance tracking, and screening for adverse events. While the health promoter typically conducts these visits, other members of the clinical investigation team may perform them when needed, following training provided by the health promoter. Each visit includes cardiometabolic monitoring, motivational support, and guidance on individualized care adjustments. All bimonthly visits—including cancellations—are documented, and each consultation is categorized by focus area to support the evaluation of adherence and trial fidelity.

### Trial status

At the time of the first manuscript submission, research ethics approval had been obtained for the trial. Participant enrollment began on 27 February 2025 at PC, the site of the coordinating investigator. Enrollment subsequently commenced at PH [27 February 2025], PM [5 March 2025], PVV [6 March 2025], PMÖ [16 September 2025], and PNO [17 September 2025]. Recruitment across all sites is expected to be completed by December 2026.

## Discussion

This investigator-initiated trial evaluates the effectiveness of LAGOM—a multicomponent, real-world intervention—in reducing cardiometabolic risk among individuals with psychotic disorders over 36 months. The core hypothesis is that a pragmatic, personalized program integrated into routine psychiatric care can lead to clinically meaningful improvements in cardiometabolic health. Delivered through outpatient psychosis clinics in the Greater Gothenburg region, LAGOM comprises five key components: (1) individualized, holistic cardiometabolic risk assessment that goes beyond isolated risk factor management; (2) structured education for patients, relatives, and staff; (3) risk-oriented referrals to primary care to address underdiagnosis and undertreatment of cardiometabolic concerns; (4) the use of visual motivational tools to strengthen patient engagement; and (5) regular follow-ups to reinforce continuity and adherence.

LAGOM was developed in response to persistent challenges in both research and clinical settings, particularly the underdiagnosis and undertreatment of cardiometabolic risk among individuals with psychotic disorders. Rather than creating new services, the intervention reorients existing workflows—such as CM follow-ups and patient education—toward systematic management of cardiometabolic and lifestyle-related health risks. Cardiometabolic risk is mapped using methods already employed in psychiatric care, and referrals align with regional protocols. Education sessions, previously focused on psychosis, are adapted to include lifestyle and cardiometabolic health. Similarly, staff training builds on existing professional development structures. The intervention fits within current roles, ensuring both scalability and sustainability. Personalized and integrated into daily practice, LAGOM aims to enhance early risk detection, patient engagement, and team capacity, potentially reducing morbidity, premature mortality, and healthcare costs. If superior to usual care, LAGOM has the potential to serve as a scalable model for addressing cardiometabolic health in one of mental healthcare’s most underserved and high-risk populations.

A central theoretical premise is that CVD risk factors interact synergistically rather than additively. Prior work has shown that combined risk factors have greater-than-additive effects on subclinical atherosclerosis and acute myocardial infarction risk. For example, Golden et al. reported that the combined effect of hypertension, hypertriacylglycerolemia, hyperinsulinemia, low HDL-C, and hyperglycemia resulted in an excess carotid intimal-medial thickness of 71 μm—compared with an expected additive increase of only 55 μm—indicating a synergistic impact on subclinical atherosclerosis [38]. Similarly, the INTERHEART study found that the combination of smoking, diabetes, hypertension, elevated apoB/apoA1 ratio, and abdominal obesity increased the odds of acute myocardial infarction to 68.5, as opposed to an additive prediction of just 12.1 [39]. This understanding—of synergistic rather than additive effects—forms one of the conceptual foundations of the LAGOM intervention and is particularly relevant for individuals with psychotic disorders, where metabolic syndrome and co-occurring CVD risk factors are highly prevalent [40]. Risk estimation tools such as SCORE2, along with monitoring whether an individual meets the criteria for metabolic syndrome, reflect this synergistic model by integrating multiple interacting risk factors into a single contextualized estimate. These approaches provide clinically meaningful ways to communicate overall cardiometabolic risk and facilitate shared understanding and decision-making between healthcare professionals and patients.

Motivation is the second cornerstone of LAGOM intervention, grounded in the capability, opportunity, motivation–behavior (COM-B) model and self-determination theory (SDT). The intervention targets reflective motivation while supporting autonomy, competence, and relatedness [41, 42]. Education for patients and relatives clarifies the link between psychosis, lifestyle behaviors, and cardiometabolic risk, helping reduce resistance and build intentions by explaining *why* change matters [41]. Parallel staff training reinforces the rationale behind interventions, supporting both engagement and professional purposes.

LAGOM operationalizes SDT through concrete practices: competence is supported via small goals accepted by patients, tangible health metrics (e.g., laboratory results, physical examination results), and structured education; relatedness is fostered through warm, supportive clinical interactions and shared understanding; and autonomy is encouraged through choice-driven changes and non-coercive sharing of rationale. The evidence suggests that when people feel capable and understand the benefits of a behavior—key aspects of empowerment—internal motivation and long-term adherence improve [42, 43]. This foundation enables patients not only to understand the consequences of unhealthy habits but also to take ownership of change. The intervention draws from the “small steps” approach, where tiny, sustainable changes create behavioral momentum and long-term identity shifts [44, 45]. This behavioral strategy emphasizes small, concrete, and achievable actions—such as walking for five minutes instead of aiming for a full workout or reducing smoking by one cigarette rather than quitting abruptly—to build self-efficacy and foster early success [44, 45]. LAGOM intentionally avoids pressuring participants with population-level guidelines; instead, it makes behaviors more achievable by breaking them down and anchoring them in personally meaningful goals.

To complement the motivational work, two visual and tangible tools—QRISK3 and the TANITA body composition analyzer—are included as exploratory supports for shared understanding and direct feedback. Although their motivational effect is not yet firmly established in this population, these tools may assist individuals with impaired executive functioning by simplifying complex health information, visually illustrating cardiometabolic risk, and providing structure to conversations between patients and healthcare professionals. QRISK3, in particular, offers a comprehensive risk assessment that highlights how modest improvements across multiple domains may yield meaningful long-term benefits. The integration of these tools builds on successful local experience with feedback-informed care, where a digital dashboard facilitated co-production between patients and healthcare professionals and enhanced patient engagement in health monitoring [46].

### Strengths and limitations

This trial has several strengths that enhance its clinical relevance, feasibility, and potential for real-world impact. It is grounded in well-established behavioral frameworks (COM-B and SDT) while being adapted to local workflows and organizational conditions in psychiatric outpatient care. Importantly, LAGOM targets both patients and healthcare professionals—an uncommon dual approach that supports sustained behavior change, professional engagement, and intervention fidelity—while also addressing the well-documented gap in awareness of the links between psychosis, lifestyle, and cardiometabolic health through structured education.

Unlike many previous trials that were short-term, narrowly focused, or dependent on resource-intensive strategies, LAGOM is designed as a long-term (36-month), scalable, and sustainable intervention integrated into routine psychiatric services. It emphasizes comprehensive, individualized cardiometabolic risk management rather than targeting isolated risk factors or single lifestyle behaviors. Broad inclusion criteria improve generalizability and ensure applicability to the diverse patients seen in everyday clinical practice. By repurposing existing roles and responsibilities—such as CM follow-up, referral pathways to primary care, and patient education—the intervention is achievable within standard patient-to-staff ratios and resource constraints. It also facilitates early detection and follow-up of cardiometabolic conditions by strengthening collaboration with primary care, addressing underdiagnosis and undertreatment.

The intervention is ecologically valid, as it operates within existing care structures without adding external personnel. Its personalized approach, which is aligned with each participant’s motivation, values, and readiness for change, enhances relevance and engagement. Contamination between groups is unlikely due to clear staff role separation, and the cluster design supports implementation fidelity across sites.

Some limitations warrant acknowledgment. The nonrandomized, cluster-assigned design introduces risks of selection bias, allegiance bias, and residual confounding. To mitigate these concerns, outcome models will include multivariable adjustments for prespecified baseline covariates (e.g., age, sex, baseline risk, and socioeconomic status). Although such adjustments cannot replace randomization or eliminate unmeasured confounding, they improve internal validity and strengthen interpretability.

Outcome data are collected by clinical staff who are involved in patient care and, at intervention clinics, in delivering the intervention. As blinding is not feasible in this pragmatic design, this may introduce a risk of measurement bias. To mitigate this, standardized data collection procedures and objective outcome measures are used across all sites.

Intervention sites were selected based on staff involvement, whereas control clinics continued with usual care. Although this limits causal inference, it enhances feasibility and reflects the complexities of real-world care. Rather than isolating individual variables—which is often neither feasible nor meaningful in this context—LAGOM assesses the combined effect of interrelated components embedded in routine practice. This approach provides insight into associations rather than isolated efficacies.

While all participating clinics include research personnel within routine clinical roles, the clinical investigation team comprises the full multidisciplinary staff at each site (including members of the research team), reflecting the pragmatic and naturalistic design of the trial. However, the intervention is delivered only at the intervention clinics and includes structured staff education and additional implementation components. These elements are integral to the intervention and aim to enhance staff engagement and implementation fidelity. As such, differences in implementation context—rather than differences in research involvement per se—may influence outcomes and limit generalizability.

Additionally, clinic-level differences—including variations in patient sociodemographic characteristics and staffing structures (e.g., staff experience and presence of trainees)—were not controlled for in the trial design and may have influenced outcomes.

Participants receive travel reimbursement for attending bimonthly physical examinations and annual education sessions as part of the trial. Such reimbursement may not always be available in routine care settings and should therefore be considered when interpreting the scalability of the intervention.

A subtle limitation is that standardizing data collection via worksheets may unintentionally influence practice. Although protocols remain unchanged, the act of documenting cardiometabolic questions could prompt more proactive management.

In summary, while LAGOM’s design limits causal inference, it supports feasibility testing, strengthens fidelity, and aligns with everyday clinical practice. The trial offers important insights into embedding structured lifestyle interventions within health services for a high-risk population. If superior to usual care, LAGOM may improve quality of life, reduce premature mortality, and provide a scalable, practice-embedded model for cardiometabolic prevention within routine psychiatric care.

**Sponsor**: Vastra Gotaland Region

**Sponsor representative and Coordinating Investigator**: Hemen Najar

Department of Psychotic Disorders, Psykosmottagning Centrum, Kronhusgatan 2G, plan 4, 411 13 Göteborg, Sweden

Email: hemen.najar@gu.se

## Supplementary Information

Below is the link to the electronic supplementary material.

Supplementary Material 1

Supplementary Material 2

## Data Availability

No datasets were generated or analysed during the current study.

## Declarations

### Dissemination policy

The trial is registered at ClinicalTrials.gov, and summary-level results will be submitted for publication in peer-reviewed scientific journals. The first results are expected to be available in 2027. Findings will also be shared at national and international conferences, through meetings with regional health authorities, and via various media channels. Trial participants are informed that they may obtain trial results through the site’s PI once available. The trial also includes collaboration with the Schizophrenia Association in Gothenburg to facilitate communication of results to broader audiences. All abstracts and publications will adhere to the authorship criteria recommended by the International Committee of Medical Journal Editors (ICMJE). The full trial CIP is available at ClinicalTrials.gov (NCT06781801).

## Abbreviations

ALT: Alanine Aminotransferase
ALP: Alkaline Phosphatase
AST: Aspartate Aminotransferase
AUDIT-C: Alcohol Use Disorders Identification Test – Consumption
BMI: Body mass index
CIP: Clinical Investigation Plan
CM: Case manager
COM-B: Capability, Opportunity, Motivation – Behavior
CVD: Cardiovascular disease
DBP: Diastolic blood pressure
DMP: Data Management Plan
eCRF: Electronic Case Report Form
EDC: Electronic Data Capture
EQ-5D-5L: EQ: EuroQol (the research group that developed it); 5D: Five Dimensions of health; 5 L: Five Levels of severity for each dimension
fP-TAG: fasting plasma triacylglycerol
FIML: Full Information Maximum Likelihood
GCP: Good Clinical Practice
HbA1c: Hemoglobin A1c, also known as glycated hemoglobin
HDL-C: High density lipoprotein-cholesterol
hs-CRP: High-sensitivity C-reactive protein
ICD-10: International Classification of Diseases, Tenth Revision
ICER: Incremental cost-effectiveness ratio
ICH-GCP: International Council for Harmonisation of Technical Requirements for Pharmaceuticals for Human Use- Good Clinical Practice
ISF: Investigator Site File
LAGOM: Longitudinal Approach to Generate positive cardiometabolic health Outcomes in severe Mental illness
MAR: Missing At Random
MCAR: Missing Completely At Random
MDR: Medical Device Regulation
MI: Multiple Imputation
MNAR: Missing Not At Random
PC: Psychosis outpatient clinic Centrum
PH: Psychosis outpatient clinic Hisingen
PI: Principal Investigator
PIS: Participant Information Sheet
PM: Psychosis outpatient clinic Mölndal
PMÖ: Psychosis outpatient clinic Öster
PNO: Psychosis outpatient clinic Nordost
PVV: Psychosis outpatient clinic Väster
QALYs: Quality-Adjusted Life Years
R-ACT: Resource-group Assertive Community Treatment
REDCap: Research Electronic Data Capture
SAEs: Serious Adverse Events
SBP: Systolic blood pressure
SCORE2: Systematic COronary Risk Evaluation 2
SCRO: Scandinavian Clinical Research Organisation
SDT: Self-Determination Theory
SPIRIT: Standard Protocol Items: Recommendations for Interventional Trials
TChol: Total cholesterol
TMF: Trial Master File
WHR: Waist-hip ratio
