# Supplementary material for: Multicenter, cluster-based, superiority trial of a multicomponent lifestyle intervention versus usual care for reducing cardiometabolic risk in individuals with psychotic disorders over 36 months: the LAGOM protocol
Source: BMC Psychiatry. 2026 Jun 23;26:487. doi: 10.1186/s12888-026-08315-3 (PMC13339501; doi:10.1186/s12888-026-08315-3)
Supplement: Supplementary file 1 — Supplementary Material 1 [file 12888_2026_8315_MOESM1_ESM.docx]

Additional table 1. Contents of the worksheet

| **Section** | **Item** | **Observation period** | **Source of information** |
| --- | --- | --- | --- |
| Administrative Information | CM Name, Physician Name, Personal ID Number, Name, Age |  |  |
| Sex and Gender | Biological Sex (Female, Male), Gender Identity (Female, Male, Other, Prefer not to say) |  | F, R |
| Social and Background Information | Date of data collection |  | F, M, R |
|  | Ethnic background (country of birth: self, parents, grandparents) |  |  |
|  | Relationship status, Living situation, Household members |  |  |
|  | Having children (biological, adopted, stepchildren, or foster children) and their number |  |  |
|  | Housing type (Ordinary, Special, Other, Homeless) |  |  |
|  | Assistance received (e.g., guardian, trustee, home care, informal support) |  |  |
|  | Employment/Social Engagement Status (detailed options like open market work, sheltered employment, studies, rehabilitation, day activity, etc.) | Since the most recent annual follow-up with the case manager (or the preceding 12 months at the first assessment) |  |
|  | Sick leave (duration and degree (%), current status) | Since the most recent annual follow-up with the case manager (or the preceding 12 months at the first assessment) |  |
|  | Permanent disability or activity compensation (status, degree (%), year granted) |  |  |
|  | Education level (highest completed level), Total years of education |  |  |
| Medical History | Family history of angina or heart attack (before age 60) |  | F, M, R |
|  | Age at first psychiatric contact |  |  |
|  | Age and type of first psychiatric symptoms |  |  |
|  | Previous or current somatic disorders (selection list) |  |  |
|  | Erectile dysfunction (men), Diagnosis/treatment for ED |  |  |
|  | Aortic ultrasound (men ≥65 years) |  |  |
|  | Cervical screening attendance (Pap smear or HPV self-sampling; women 23–70 years), Mammography screening attendance (women 40–74 years) |  |  |
|  | Dental care support (need and justification) |  |  |
|  | Use of steroids (oral or parenteral) |  |  |
|  | Antipsychotic medication (drugs used, treatment purpose) |  |  |
|  | Antihypertensives (drugs used, treatment purpose) |  |  |
|  | Lipid-lowering drugs |  |  |
|  | Diabetes medication (drugs used, purpose: diabetes/obesity) |  |  |
|  | Inpatient psychiatric care (frequency, days, compulsory care count) | Since the most recent annual follow-up with the case manager (or the preceding 12 months at the first assessment) |  |
|  | ICD diagnosis code for psychotic disorder |  |  |
|  | Year of first psychosis diagnosis |  |  |
|  | Comorbid psychiatric disorders (ICD codes) |  |  |
| Lifestyle Habits | Sleep  Sleep problems and interventions (e.g., medication, weighted blanket) |  | F, R, S |
|  | Smoking  Status, start/stop age, quantity |  |  |
|  | Snus (Swedish smokeless tobacco)  Status, start/stop age, quantity |  |  |
|  | Physical Activity  Daily activities: low- to moderate-intensity (walking, shopping, chores)  Physical exercise: to breathlessness (e.g., gym, running)  Sedentary time: sleeping, napping, screen time  Total time (active + sedentary + sleep) should sum to 24h | Typical day during the last 12 months |  |
|  | Dietary Index  Frequency of vegetables, fruits, fish, sweets/snacks, breakfast; Dietary Index score (0–12) | Last 12 months |  |
|  | Alcohol (AUDIT-C)  Frequency of drinking, quantity per session, binge drinking, AUDIT-C score | Last 12 months |  |
|  | Substance Use Disorders  Including alcohol, cannabis, opiates, sedatives, tobacco, stimulants (e.g., caffeine) (per ICD F-codes) |  |  |
| Physical Examination | Date, Time, BP, Pulse, Height, Weight, Waist & Hip circumference |  | F |
|  | Body composition via TANITA body composition analyzer*: fat mass, muscle mass, water, bone mass, metabolic age |  |  |
| Blood Tests | Date/time of test  Fasting status (Yes/No) and number of fasting hours  P-CRP, P-Creatinine, eGFR, P-AST, P-ALT, P-ALP, P-Bilirubin, P-Glucose, B-HbA1c, P-Cholesterol (Total, LDL, HDL, non-HDL), P-Triacylglycerol |  | F, M |
| Assessment Scales | EQ-5D-5L  Mobility, Personal care, Usual activities, Pain/discomfort, Anxiety/depression, Self-rated health (0–100) |  | F, S |
|  | Metabolic Syndrome*  Defined by waist size, triglycerides, HDL, BP, glucose |  |  |
|  | SCORE2 & QRISK3*  CVD risk scores, Healthy heart age |  |  |
| Other Information* | Participation in LAGOM health education | Since the most recent annual follow-up with the case manager (or the preceding 12 months at the first assessment) | F, M, R |
|  | Sessions with internal/external healthcare professionals on physical activity, diet, smoking, alcohol | Since the most recent annual follow-up with the case manager (or the preceding 12 months at the first assessment) |  |
|  | Experience using TANITA and QRISK3 |  |  |
| * Only intervention clinics.  Abbreviations: B-HbA1c, Blood Hemoglobin A1c; BP, blood pressure; ED, erectile dysfunction; eGFR, estimated glomerular filtration rate; F, face-to-face interviews; M, medical records; P-ALP, Plasma Alkaline Phosphatase; P-ALT, Plasma Alanine Transaminase; P-AST, Plasma Aspartate Transaminase; P-CRP, Plasma C Reactive Protein; P-HDL, Plasma High Density Lipoprotein Cholesterol; P-LDL, Plasma Low Density Lipoprotein Cholesterol ; R, remote interviews; S, self-administered questionnaires completed at home. | | | |
