# Supplementary material for: Multicenter, cluster-based, superiority trial of a multicomponent lifestyle intervention versus usual care for reducing cardiometabolic risk in individuals with psychotic disorders over 36 months: the LAGOM protocol
Source: BMC Psychiatry. 2026 Jun 23;26:487. doi: 10.1186/s12888-026-08315-3 (PMC13339501; doi:10.1186/s12888-026-08315-3)

**Broschyr**

**LAGOM**

Ett individanpassat hälsofrämjande program för att minska den kardiometabola risken hos individer med en allvarlig psykisk sjukdom


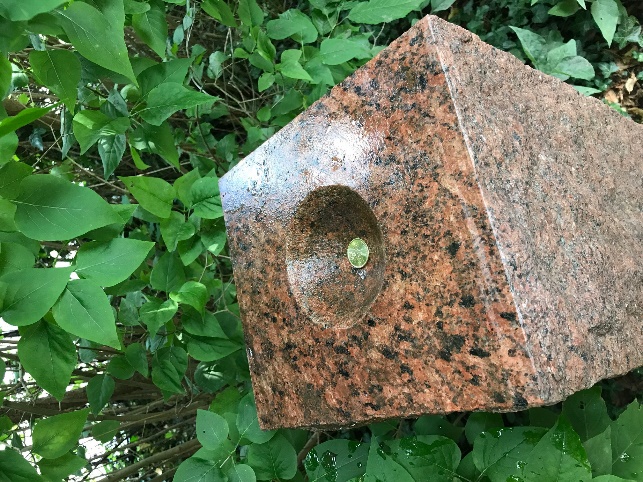

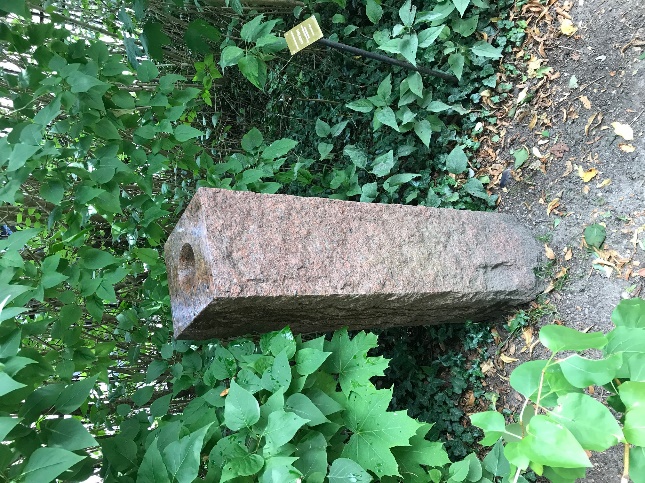

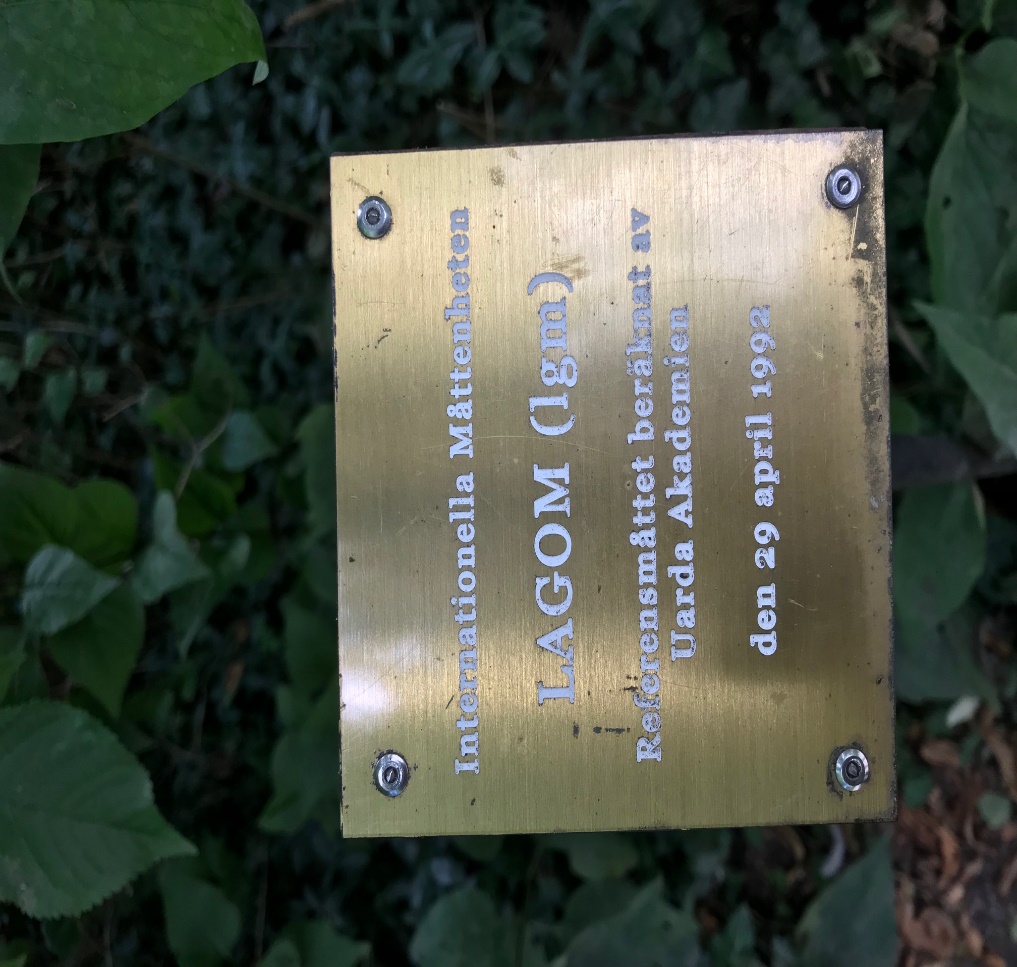


Table of Contents

[**Introduktion** 1](#_Toc212551039)

[**Avsnitt 1** 3](#_Toc212551040)

[**Vad är LAGOM?** 3](#_Toc212551041)

[**Avsnitt 2** 4](#_Toc212551042)

[**Frågor och svar** 4](#_Toc212551043)

[Vad är grundprincipen? 4](#_Toc212551044)

[Vad är huvudsyftet? 4](#_Toc212551045)

[Vilka mål har LAGOM? 4](#_Toc212551046)

[Vad är första steget? 4](#_Toc212551047)

[Hur kartläggas den kardiometabola profilen? 4](#_Toc212551048)

[Användning av Metabola syndromet och SCORE2? 5](#_Toc212551049)

[Hur kan vi identifiera och åtgärda den enskilda ohälsosamma levnadsvanan? 7](#_Toc212551050)

[Vad händer efter kartläggningen? 7](#_Toc212551051)

[Hur ofta sker uppföljningen? 7](#_Toc212551052)

[Vilka verktyg har vi i vårt motivationsarbete? 8](#_Toc212551053)

[Ingår det psykoedukation och fortbildning? 10](#_Toc212551054)

[**Avsnitt 3** 11](#_Toc212551055)

[**Några begrepp** 11](#_Toc212551056)

[Enkla råd 11](#_Toc212551057)

[Rådgivande samtal 11](#_Toc212551058)

[Kvalificerat rådgivande samtal 11](#_Toc212551059)

[Svårighetsgrad av levnadsvanorna 11](#_Toc212551060)

[**Avsnitt 4** 12](#_Toc212551061)

[**Daglig rökning** 12](#_Toc212551062)

[Definition 12](#_Toc212551063)

[Svårighetsgrad 12](#_Toc212551064)

[Föreslagen åtgärd 12](#_Toc212551065)

[Bedömning av riskbeteende – Tobaksbruk enligt Socialstyrelsen 12](#_Toc212551066)

[Länkar om rökavvänjning 12](#_Toc212551067)

[**Avsnitt 5** 13](#_Toc212551068)

[**Ohälsosamma matvanor** 13](#_Toc212551069)

[Definition 13](#_Toc212551070)

[Svårighetsgrad 13](#_Toc212551071)

[Föreslagen åtgärd 13](#_Toc212551072)

[Länkar om matvanor 13](#_Toc212551073)

[Läkemedelsbehandling 14](#_Toc212551074)

[**Avsnitt 6** 15](#_Toc212551075)

[**Riskbruk av alkohol** 15](#_Toc212551076)

[Definition 15](#_Toc212551077)

[Svårighetsgrad 15](#_Toc212551078)

[Föreslagen åtgärd 15](#_Toc212551079)

[Länkar om riskbruk av alkohol 15](#_Toc212551080)

[**Avsnitt 7** 16](#_Toc212551081)

[**Otillräcklig fysisk aktivitet** 16](#_Toc212551082)

[Definition 16](#_Toc212551083)

[Svårighetsgrad 16](#_Toc212551084)

[Föreslagen åtgärd 16](#_Toc212551085)

[Länkar om rådgivning och hänvisning till fysisk aktivitet 17](#_Toc212551086)

[**Avsnitt 8** 19](#_Toc212551087)

[**Bilagor** 19](#_Toc212551088)

# **Introduktion**

Individer med allvarlig psykisk sjukdom som psykos sjukdom, egentlig depression, och bipolära syndrom drabbas av kardiometabola sjukdomar som hjärtkärlsjukdom, fetma, och diabetes mellitus typ II i större utsträckning jämfört med den allmänna befolkningen. En av konsekvenserna till hjärtkärlsjukdom är för tidig död. Individer med allvarlig psykisk sjukdom har 10 till 20 år kortare livslängd jämfört med den allmänna befolkningen. Förutom detta, har individerna med allvarlig psykisk sjukdom sämre livskvalitet med ökad sjukfrånvaro, och för tidig pension på grund av konsekvenserna till de kardiometabola sjukdomarna. Samhället är också påverkat på grund av förlust av produktion samt pga. de ökade utgifterna för behandling av individer med allvarlig psykisk sjukdom.

Det finns olika riskfaktorer som orsakar den ökade risken för att drabbas av dessa kardiometabola sjukdomar. Dessa riskfaktorer är, delvis, själva psykiska sjukdomen och dess genetiska benägenhet, och, delvis, yttre faktorer som behandling med psykofarmaka, ohälsosamma matvanor, otillräcklig fysisk aktivitet, tobaksbruk, och riskbruk av alkohol, samt den sämre kvaliteten på den vård som individer med allvarlig psykisk sjukdom får jämfört med den allmänna befolkningen.

Många av dessa riskfaktorer kan modifieras genom val av psykofarmaka, och genom hjälp mot de olika ohälsosamma levnadsvanorna. För att kunna erbjuda hjälp till personer i behov av stöd att förändra sina ohälsosamma levnadsvanor behöver vårdpersonalen ställa frågor om dessa. Befolkningsundersökningar visar att patienterna tycker att det är positivt att läkare eller annan vårdpersonal diskuterar levnadsvanor vid besök i vården.

Socialstyrelsen har, år 2018, kommit ut med nationella riktlinjer för prevention och behandling vid ohälsosamma levnadsvanor. Dessa riktlinjer bygger på fyra pelare:

- Daglig rökning/snusning
- Ohälsosamma matvanor
- Riskbruk av alkohol
- Otillräcklig fysisk aktivitet

I de centrala rekommendationerna betonar Socialstyrelsen särskilt rekommenderade åtgärder för att förändra de ohälsosamma levnadsvanorna hos, bland andra, vuxna med särskild risk och som exempel på sådana grupper är personer som har:

- En sjukdom (till exempel diabetes, astma, KOL, cancer, hjärt-kärlsjukdom, lång-varig smärta, schizofreni eller depression).
- Fysisk, psykisk eller kognitiv funktionsnedsättning.
- Social sårbarhet (till exempel låg socioekonomisk ställning).
- Biologiska riskmarkörer (till exempel högt blodtryck, blodfettsrubbningar, övervikt eller fetma).
- Andra riskfaktorer (till exempel flera ohälsosamma levnadsvanor samtidigt eller en olycksfallsskada).

Individer med allvarlig psykisk sjukdom tillhör flera av kategorierna ovan gällande vuxna med särskild risk. Till skillnad från vuxna generellt i den allmänna befolkningen, har individer med allvarlig psykisk sjukdom en ökad sårbarhet för stress. Denna sårbarhet ställer krav på oss inom sjukvården att anpassa förändringsåtgärderna gällande de ohälsosamma levnadsvanorna hos dessa individer till en lagom nivå (därmed namnet ”LAGOM”), för att öka förutsättningarna att individen kan följa vårdpersonalens råd och att förändringarna är giltiga på lång sikt.

Enligt Socialstyrelsen är det vetenskapliga underlaget om effekterna av åtgärderna för vuxna med särskild risk i de flesta fall otillräckligt. Prioriteringen för denna grupp har därför huvudsakligen utgått från underlaget för vuxna generellt, och är genomgående högre än för vuxna generellt på grund av den högre svårighetsgraden.

I Socialstyrelsens rapport "Nationell utvärdering av vård och stöd vid schizofreni och schizofreniliknande tillstånd –2022 Huvudrapport med förbättringsområden" står i sammanfattningen att "För att fortsatt motverka ohälsa och en överdödlighet i gruppen behöver omhändertagandet inom såväl psykiatrisk som somatisk vård och förebyggande vård fortsatt utvecklas. Viktiga instrument här är årliga fysiska hälsokontroller som innefattar hälsosamtal, somatisk undersökning och provtagning samt screening för att identifiera fysisk ohälsa och sjukdom. Vid kända riskfaktorer som till exempel viktuppgång, ärftlighet för hjärt- och kärlsjukdom eller diabetes är det nödvändigt med tätare uppföljning.".

Vidare står det i samma sammanfattning att "Denna utvärdering visar att en del av rekommendationerna i riktlinjerna har fått genomslag i verksamheterna. Exempel på detta är årskontroller av somatisk hälsa tillsammans med rådgivande samtal kring hälsosamma levnadsvanor. Däremot är det svårare för vården att erbjuda ledarledda insatser kopplade till fysisk aktivitet och kost, vilket behöver utvecklas.".

# **Avsnitt 1**

# **Vad är LAGOM?**

LAGOM är ett kunskapsbaserat, mångfacetterat, och hälsofrämjande program för att hjälpa individen att skaffa sig en bättre livskvalitet, ha en bättre kardiometabol profil, och ha hälsosamma levnadsvanor. LAGOM avser att implementera Socialstyrelsens rekommendationer och riktlinjer avseende individernas levnadsvanor med anpassning efter individens samt efter psykosklinikens arbetssätt. LAGOM är inspirerat av kognitiv beteendeterapi genom sin tonvikt på att skapa en bättre förståelse av förhållandet mellan den allvarliga psykiska sjukdomen, den kardiometabola profilen, och levnadsvanorna. LAGOM erbjuder en livsstilscoaching och avser att hjälpa individer med allvarlig psykisk sjukdom genom att ha bättre kommunikation med primärvården och hitta individuellt anpassade livsstilar vad gäller kostvanor, fysisk aktivitet, alkoholvanor, samt tobaksbruk. De övergripande syften till LAGOM är att:

- Erbjuda ett jämlikt, relevant, och effektivt program som ska spegla god vård och omsorg.
- Förbättra de kardiometabola avvikelserna som vikt, blodfetter (triacylglycerol (TAG) (tidigare kallat triglycerid), total kolesterol, HDL-kolesterol, och LDL-kolesterol), glukos och blodtrycket hos deltagande individer.
- Nå individer med långvarig psykiatrisk ohälsa med hälsofrämjande och förebyggande insatser.
- Identifiera riskfaktorer för diabetes mellitus typ II och kardiovaskulära sjukdomar hos individer med långvarig psykiatrisk ohälsa.
- Skapa förutsättningar för att minimera risken att drabbas av hjärtkärlsjukdomar eller diabetes.
- Höja deltagarnas kunskap och medvetenhet om sina levnadsvanor och sin hälsa genom att använda objektiva mått på de olika levnadsvanorna, samt mått på de olika kardiometabola avvikelserna, med hjälp av blanketter, blodprov, klinisk undersökning, samt riskalgoritmer för hjärtkärlsjukdomar.
- Regelbundet följa upp individerna som deltar i programmet för att stödja deras tillämpning av rekommendationerna.
- Objektivt mäta effekten av insatserna med samma objektiva mått som ovan samt effekten på livskvaliteten och den psykiska och fysiska hälsan i övrigt.

Fyra aspekter är viktiga i detta program:

1. Att fokusera på att förbättra livskvaliteten och förebygga kardiometabola sjukdomar.
2. Att genom riskalgoritmer för hjärtkärlsjukdomar utvärdera riskfaktorer för kardiometabola sjukdomar samlat i ett sammanhang i stället för var och en av faktorerna isolerat.
3. Att ha i åtanke att individer med allvarlig psykisk sjukdom har en ökad stresskänslighet och därmed inte kan ta till sig informationen på samma sätt som vuxna i den allmänna befolkningen. Därav är det viktigt att åtgärderna är individanpassade och på en lagom nivå.
4. Att följa upp deltagarna både när de får hjälp från psykoskliniken och när de får extern hjälp, samt före och efter insatsen.

# **Avsnitt 2**

# **Frågor och svar**

## Vad är grundprincipen?

Grundprincipen är en förändring på sikt, i rätt riktning och i en lagom takt som är individanpassad. Till exempel: Den person som inte rör på sig ska börja röra på sig mer första året och mer följande åren. Den som röker 20 cigaretter om dagen ska röka mindre första året och mindre följande åren. Samma princip gäller för kostvanor och alkoholvanor. Att gå ner i vikt är en stor utmaning som individer med allvarlig psykisk sjukdom har. Viktnedgång behöver inte stå i centrum för våra insatser. Flera studier har visat att riskökningen är ett resultat av en samling av flera riskfaktorer. Om vi lyckas hjälpa våra patienter att förbättra några andra riskfaktorer när de inte kan gå ner i vikt så har vi lyckats minska risken för kardiometabola sjukdomar och dessutom förbättrat livskvaliteten hos dessa individer.

## Vad är huvudsyftet?

Att höja livskvaliteten, förbättra den psykiska och fysiska hälsan, samt minska risken för funktionsnedsättningar och kardiometabola sjukdomar; diabetes mellitus typ II och hjärtkärlsjukdomar (stroke, transitorisk ischemisk attack, hjärtinfarkt, eller angina pectoris).

## Vilka mål har LAGOM?

- Att förbättra den kardiometabola profilen hos individer med allvarlig psykisk sjukdom.
- Att stoppa den försämrade trenden av för tidig död hos individer med allvarlig psykisk sjukdom.
- Att öka kunskap hos patienter och vårdpersonal om sambandet mellan den psykiska sjukdomen, kardiometabola profilen, och levnadsvanorna.
- Att minska sjukvårds- och samhällskostnader kopplade till behandling av individer med allvarlig psykisk sjukdom.

## Vad är första steget?

Första steget är en kartläggning av den kardiometabola profilen och levnadsvanorna enligt flödesschemat (bilaga 1) vid årskontrollen.

## Hur kartläggas den kardiometabola profilen?

Den kardiometabola profilen analyseras genom följande:

1. **Samsjuklighet eller metabolt syndrom**
   Kartläggning av samtidig diabetes mellitus eller uppfyllande av kriterierna för metabola syndromet.
2. **Riskbedömning enligt SCORE2**
   Bedömning av risken för hjärt-kärlsjukdom med hjälp av riskalgoritmen SCORE2:
   - Hög risk: SCORE2 ≥ 2,5 % (<50 år) eller ≥ 5 % (≥50 år)
   - Mycket hög risk: SCORE2 ≥ 7,5 % (<50 år) eller ≥ 10 % (≥50 år)
3. **Utveckling av kardiometabola parametrar över tid**
   Övervakning av förändringar i kardiometabola parametrar såsom:
   - Blodprover: triglycerider (TAG), HDL-kolesterol, LDL-kolesterol, totalt kolesterol, glukos
   - Viktparametrar: midjemått, BMI
   - Blodtryck
     *(Observera att det idag saknas en etablerad konsensus kring exakta gränser för vad som räknas som en kliniskt signifikant försämring.)*
4. **Avvikande laboratorievärden**
   Identifiering av avvikande värden baserat på laboratoriets aktuella referensintervall och cut-off-gränser.
5. **Ärftlighet för hjärt-kärlsjukdom**
   Förekomst av hjärt-kärlsjukdom hos förstagradssläkting före 60 års ålder.

## Användning av Metabola syndromet och SCORE2?

Vid bedömning av kardiometabola avvikelser är det viktigt att analysera de olika parametrarna i ett samlat sammanhang, snarare än att utvärdera varje faktor isolerat. Detta eftersom det finns ett synergistiskt samband mellan avvikelserna och deras påverkan på risken för hjärt-kärlsjukdomar.

Ett praktiskt och strukturerat sätt att identifiera kardiometabola avvikelser är att utgå från definitionen av det metabola syndromet enligt tabeller 1 och 2 nedan.





Borrowed with permission from Alberti KGMM et al. Harmonizing the metabolic syndrome. Circulation. 2009;120:1640-1645.





Borrowed with permission from Alberti KGMM et al. Harmonizing the metabolic syndrome. Circulation. 2009;120:1640-1645.

Om individen uppfyller tre av dessa fem kriterier betraktas hen som att ha metabola syndromet.

SCORE2 är en prediktionsalgoritm som används inom hälso- och sjukvården i Sverige för att uppskatta risken för hjärt-kärlsjukdom (CVD).
Alla europeiska länder har grupperats i fyra riskregioner baserat på deras senaste WHO-standardiserade dödlighet i hjärt-kärlsjukdom per 100 000 invånare (ICD-10 kapitel IX, I00–I99):

- Låg risk (<100 CVD-dödsfall per 100 000) (modell A)
- Måttlig risk (100 till <150 CVD-dödsfall per 100 000) (modell B)
- Hög risk (150 till <300 CVD-dödsfall per 100 000) (modell C)
- Mycket hög risk (≥300 CVD-dödsfall per 100 000) (modell D)

Eftersom individer med psykossjukdom har en högre CVD-dödlighet än 300 dödsfall per 100 000 invånare, tillhör dessa individer en mycket hög riskgrupp.
För denna patientgrupp är det därför mer lämpligt att använda modell D av SCORE2, enligt nedan.

(Tabellerna nedan är adapterade från *European Heart Journal*, Volume 42, Issue 25, 1 July 2021, Pages 2439–2454, 2455–2467)


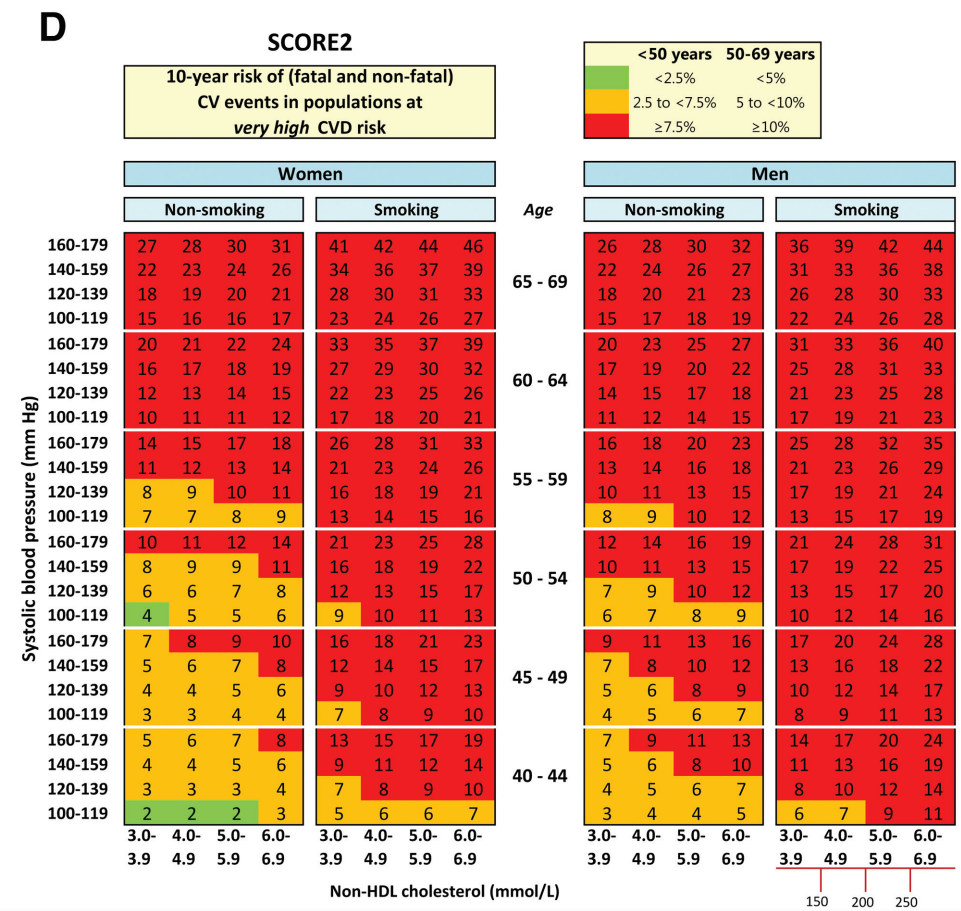


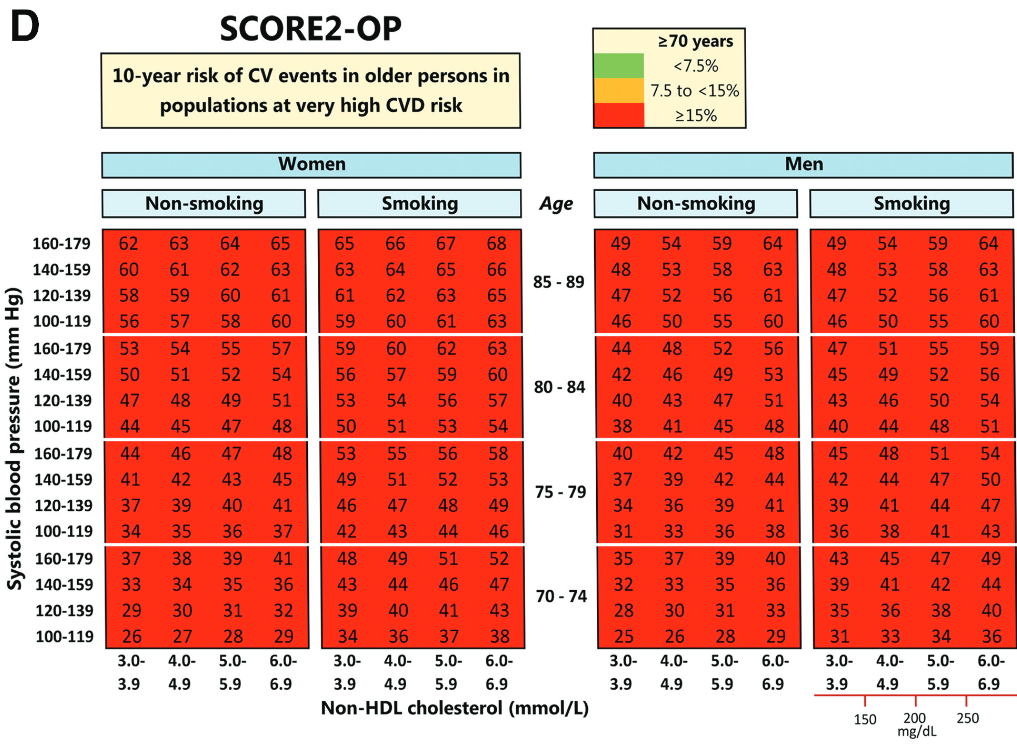


## Hur kan vi identifiera och åtgärda den enskilda ohälsosamma levnadsvanan?

Definition och förslag på åtgärd finns i avsnitt 4–7 nedan.

## Vad händer efter kartläggningen?

Efter att ohälsosamma levnadsvanor eller avvikelser i den kardiometabola profilen har identifierats vid årsbesöket, gör den ansvariga läkaren en medicinsk bedömning för att överväga remittering till externa aktörer, såsom primärvården, som kan erbjuda behandling för kardiometabola avvikelser. Detta kan inkludera blodfettsänkande behandling, blodtryckssänkande behandling, diabetesmedicinering eller behandling av fetma. Primärvården kan även tillhandahålla insatser såsom kostrådgivning och stöd för rökavvänjning.

Den ansvariga läkaren kan också remittera patienten till interna aktörer, såsom hälsopedagoger och fysioterapeuter, för vidare utvärdering av patientens resurser och motivation att förändra avvikelserna i den kardiometabola profilen.

Case managern har ansvar för att följa upp både interna och externa insatser på ett individanpassat sätt genom regelbundna kontakter och uppföljningar under året. Eventuella komplexa ärenden diskuteras vid behandlingskonferensen.

## Hur ofta sker uppföljningen?

Uppföljningen sker individuellt med jämna mellanrum under året för att inte tappa effekten av de olika insatserna. Uppföljningen ska vara individanpassad och kommer att ske oftare i början tills patienten har hittat ett fungerande sätt att hantera sina kardiometabola avvikelser och sina ohälsosamma levnadsvanor.

Ett exempel på dessa fungerande sätt kan vara:

- Etablering av en fungerande kontakt med en dietist på vårdcentralen för bättre kostvanor.
- Förskrivning av läkemedel mot kardiometabola avvikelser av primärvårdsläkaren.
- Etablering av en fungerande kontakt med en distriktsjuksköterska för rökavvänjningen.
- Behärskandet av de olika länkarna till hälsoteket, friskvårdsklubben, samt de olika apparna om levnadsvanorna.
- Etablering av en fungerande kontakt med hälsopedagogen för bättre levnadsvanor.

## Vilka verktyg har vi i vårt motivationsarbete?

Samtalsmetoden MI kan utgöra en användbar grund för hälsosamtalen. Att motivera kan innefatta att ge individen konkreta och individanpassade skäl till att personen bör ändra sina levnadsvanor. QRISK3 kan användas i detta syfte. QRISK3 ger ett tydligt exempel på det interaktiva förhållandet mellan olika riskfaktorer och ger en vägledning när det gäller förändring av de modifierbara riskfaktorerna. Därmed kan den också vara användbar i motivationsarbetet, se nedan! Det bör dock noteras att QRISK3-skalan inte är validerad för användning inom den svenska populationen eller för individer med allvarlig psykisk sjukdom. Här nedan följer en beskrivning med bilder av hur QRISK3-skalan ser ut och hur den kan tillämpas.


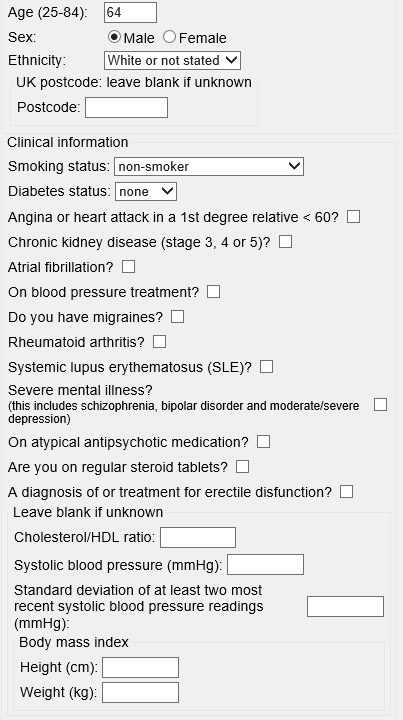


Under ”Ethnicity” kan följande alternativ väljas:


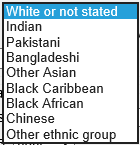


Under ”Smoking status” kan man välja följande alternativ:


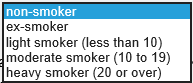


Under ”Diabetes status” finns följande alternativ:


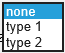


De modifierbara riskfaktorerna är:

Rökning, total kolesterol/HDL kvot, systoliskt blodtryck, och BMI.

De ”delvis” modifierbara riskfaktorerna är:

Behandling med atypiska antipsykotiska.

Nedan visas ett exempel på en 40-årig man med psykossjukdom:


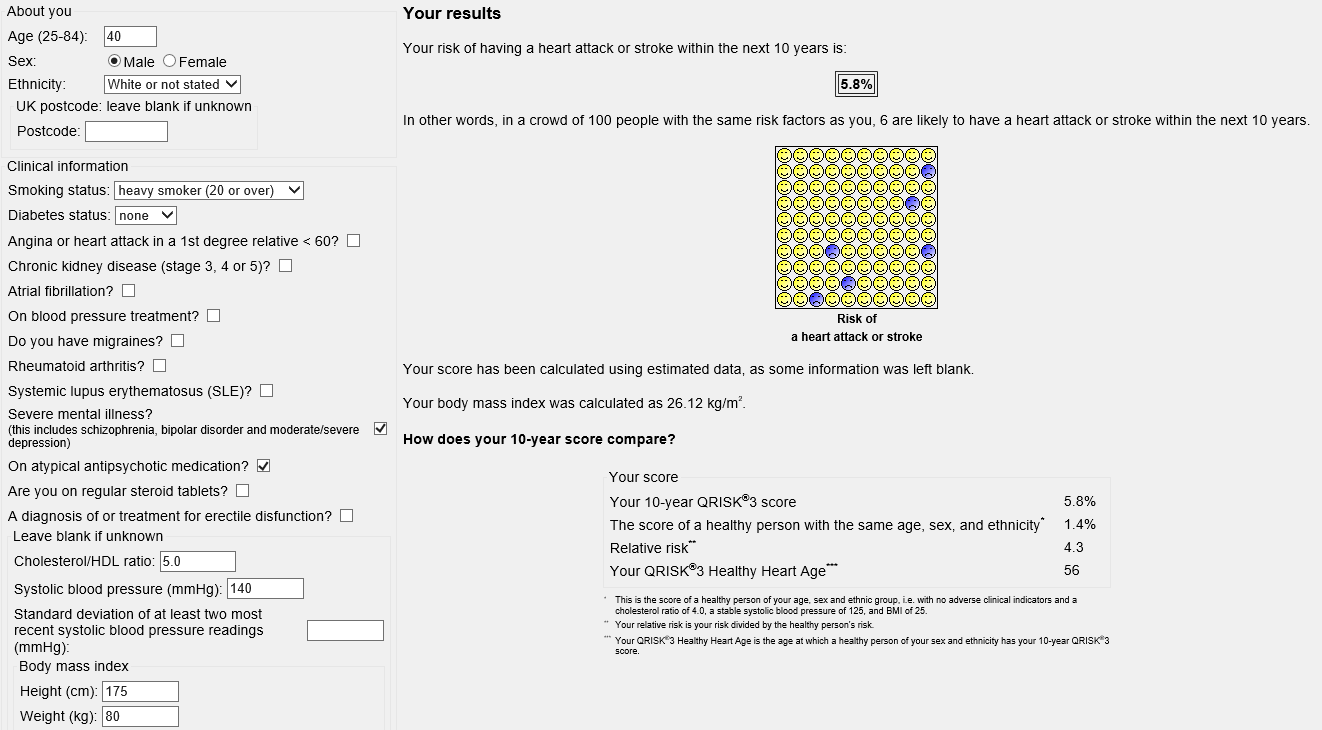


Denna individ har en absolut risk på 5,8 %. För att förstå denna procentsiffra kan vi jämföra med procentsiffran 1,4 % som tillhör en person av samma ålder, kön, och etnicitet som denna individ. Här ser vi den stora skillnaden. Vi ser dessutom att denna 40-åriga man har ett hjärta som har en högre biologisk ålder (56 år) jämfört med dess kronologiska ålder (40 år).

Om vi lyckas att få denna individ att sluta röka kommer han i stället att få dessa siffror:


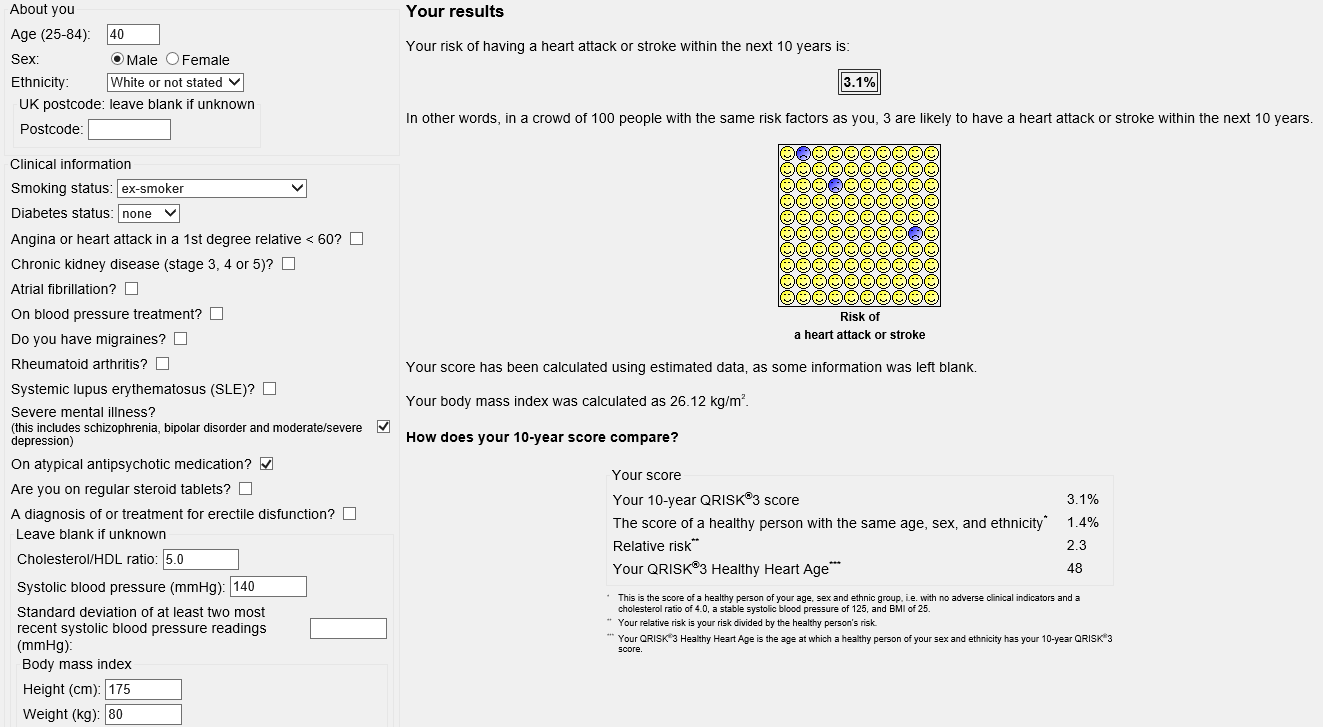


Därför kan QRISK3 och andra riskskalor ha ett pedagogiskt budskap genom möjligheten att hypotetiskt ändra de olika modifierbara riskfaktorerna och visa hur utfallet kan förändras!

## Ingår det psykoedukation och fortbildning?

LAGOM inkluderar årlig psykoedukation för personer med allvarlig psykisk sjukdom om sambandet mellan den kardiometabola profilen, den psykiska sjukdomen och levnadsvanor. LAGOM innehåller även fortbildning för medarbetare om programmet genom interna seminarier som planeras att hållas två gånger årligen.

# **Avsnitt 3**

# **Några begrepp**

## Enkla råd

Socialstyrelsen ger inga rekommendationer angående användningen av åtgärden enkla råd, vilket innebär information och kortfattade standardiserade råd om levnadsvanor. Det är dock av betydelse att hälso- och sjukvården fortsätter att erbjuda enkla råd om levnadsvanor. Enkla råd utgör en naturlig del av bedömningssamtal som genomförs av hälso- och sjukvården för att identifiera och uppmärksamma ohälsosamma levnadsvanor hos patienter. Dessa råd bör betraktas som en grund för påföljande behandling med mer omfattande åtgärder, såsom rådgivande eller kvalificerade rådgivande samtal, och bör kunna ges på alla nivåer inom hälso- och sjukvården.

## Rådgivande samtal

En dialog som fokuserar på individen, anpassad till den specifika personens ålder, hälsotillstånd, risknivåer, med mera. Det kan inkludera motiverande strategier såsom motiverande samtal och kan stärkas med olika verktyg och hjälpmedel. Åtgärden kan också kompletteras med återkommande kontakter, såsom återbesök, telefonsamtal, brev eller mejl, vid ett eller flera tillfällen. Normalt sett tar åtgärden 5–15 minuter, men kan i vissa fall sträcka sig upp till cirka 30 minuter.

## Kvalificerat rådgivande samtal

En individanpassad dialog med patienten, skräddarsydd efter den specifika individens ålder, hälsotillstånd, risknivåer med mera, kan inkludera motiverande strategier, såsom motiverande samtal, och kan kompletteras med olika verktyg och hjälpmedel. Åtgärden är teoribaserad och strukturerad, vilket innebär att den bygger på tydligt definierade antaganden om hur och varför den fungerar och inkluderar fördefinierade komponenter. För att genomföra åtgärden krävs att personalen har djupgående kunskap inom ämnet och är utbildad i den metod som används för samtalet. Den tar mer tid i anspråk jämfört med rådgivande samtal, och återkommande sessioner eller kontakter (återbesök, telefonsamtal, brev eller mejl) kan äga rum vid ett eller flera tillfällen. Åtgärden kan genomföras individuellt eller i grupp och omfattar ofta komponenter från olika teorier, såsom Social learning theory, Social cognitive theory, Health belief model, Theory of planned behavior, Stages of change and Transtheoretical model, Cognitive behavior theory, Self-determination theory, och Protection motivation theory. Kvalificerade rådgivande samtal kan kräva särskilda utbildningsinsatser.

## Svårighetsgrad av levnadsvanorna

För varje tillstånd i riktlinjerna har Socialstyrelsen gjort en bedömning av tillståndets svårighetsgrad. Bedömningen av svårighetsgraden har i dessa riktlinjer utgått från risken för framtida ohälsa.

Socialstyrelsen har utgått ifrån svårighetsgraden för ett antal allvarliga sjukdomar som är förknippade med respektive levnadsvana och risken att drabbas av dessa sjukdomar till följd av den ohälsosamma levnadsvanan om ingen åtgärd sätts in. För att uppskatta risken att drabbas av dessa sjukdomar har Socialstyrelsen tagit utgångspunkt i den samlade sjukdomsbördan.

# **Avsnitt 4**

# **Daglig rökning**

## Definition

Med daglig rökning avses rökning dagligen, oavsett mängd cigaretter. För gravida, unga under 18 år och personer som ska opereras inkluderas även mer sporadisk rökning. All rökning, även tillfällig rökning och i små mängder, är förenad med hälsorisker, men risken ökar med antalet cigaretter man röker och hur länge man har rökt.

Enligt Socialstyrelsen:

- Vid bedömning av riskbeteende utgå från resultat av bedömningsinstrumenten, patientens hälso- och sjukdomstillstånd och samtal med patienten.
- För vissa sjukdomstillstånd är de allmänna råden inte förenliga med de sjukdomsanpassade råden.
- Patienten ska erbjudas råd och stöd utifrån sin situation och sitt individuella behov.
- Råd och information om riskbeteende bör vara kopplat till patientens hälsotillstånd och sjukdom samt till de vinster patienten gör med att sluta med sin ohälsosamma levnadsvana.

## Svårighetsgrad

Stor till mycket stor.

## Föreslagen åtgärd

Kvalificerat rådgivande samtal. Rökavvänjning i grupp hos hälsopedagogen, hänvisa till vårdcentralen eller se länkar nedan! (OBS! Glöm inte grundprincipen i avsnitt 2 på sida 4!).

## Bedömning av riskbeteende – Tobaksbruk enligt Socialstyrelsen

Riskbeteende föreligger vid:

- All daglig rökning hos vuxna

- Sporadisk rökning hos vuxna som ska eller genomgått operation eller hos högriskgrupper*

- Rökning hos ungdomar under 18 år

- Daglig snusning

* Högriskgrupper är exempelvis individer med högt blodtryck, blodfettsrubbningar, övervikt eller fetma, lungsjukdom, cancer, diabetes, hjärtkärlsjukdom, schizofreni, depression, ammande och föräldrar/vårdnadshavare till små barn.

Om individen uppger annat tobaksbruk än cigarett eller snus, ex cigarr, vattenpipa eller e-cigarett ska detta bedömas och dokumenteras i patientjournalen.

## Länkar om rökavvänjning

1. Vårdgivarwebben

<https://www.vgregion.se/halsa-och-vard/vardgivarwebben/vardriktlinjer/levnadsvanor-och-sjukdomsforebyggande-metoder/>tobak/

Här finns information om levnadsvanan Tobak.

1. Hälsocoach online

<https://www.vgregion.se/halsa-och-vard/levnadsvanor/halsocoach-online/>

Lämpar sig för personer som har en hög grad av självständighet och motivation till förändring av levnadsvanor. Citat från hemsidan: ”Det är viktigt att personen är motiverad och kan ta ansvar för att förändra sina levnadsvanor.”

# **Avsnitt 5**

# **Ohälsosamma matvanor**

## Definition

Ohälsosamma matvanor definieras som kostmönster som inte är förenliga med att förbättra den kardiometabola profilen eller förebygga kardiometabol sjukdom.
Detta kan till exempel innebära att individen fortsätter att öka i vikt till följd av sina matvanor, vilket tyder på att kosten bidrar till en ogynnsam utveckling av riskfaktorer såsom övervikt, hyperlipidemi, hyperglykemi eller hypertoni.
Kort sagt: **om kosten förvärrar eller inte hjälper till att förbättra kardiometabola riskfaktorer betraktas den som ohälsosam**.

Socialstyrelsen har definierat betydande ohälsosamma matvanor som låga poäng på kostindexet (0–4 poäng av 12 möjliga). Utifrån denna definition har cirka 20 procent av befolkningen ohälsosamma matvanor.

## Svårighetsgrad

Stor.

## Föreslagen åtgärd

Kvalificerat rådgivande samtal. Remiss till dietist på vårdcentral, klinisk nutrition, eller obesitasmottagning beroende på grad av övervikt och medföljande komplikationer! (OBS! Glöm inte grundprincipen i avsnitt 2 på sida 4!).

Bilagor: 2 och 3.

OBS! Obesitasmottagningen tar emot vårdbegäran för personer med BMI ≥35 kg/m^2^. För fetmaoperation gäller BMI ≥40 kg/m^2^, eller BMI ≥35 kg/m^2^ med samsjuklighet (diabetes typ II, fetmarelaterad kardiomyopati, svår sömnapné, lungemboli, pseudotumör cerebri, venösa bensår), och frånvaro av kontraindikatorer. Bilaga 3 nedan visar den blankett som bör skickas med remissen!

## Länkar om matvanor

1. Vårdgivarwebben

<https://www.vgregion.se/halsa-och-vard/vardgivarwebben/vardriktlinjer/levnadsvanor-och-sjukdomsforebyggande-metoder/matvanor/>

Information om levnadsvanan Matvanor.

1. Klinisk nutrition

<https://www.sahlgrenska.se/omraden/omrade-6/specialistmedicin/enheter/dietistmottagning-klinisk-nutrition-sahlgrenska/>

1. Råd om kost

<https://www.1177.se/vastra-gotaland/liv--halsa/ata-for-att-ma-bra/sa-ater-du-halsosamt/>

<https://www.halsolots.se/>

1. Hälsocoach online

<https://www.vgregion.se/halsa-och-vard/levnadsvanor/halsocoach-online/>

## Läkemedelsbehandling

(Hämtad från VGRs MEDICINSK RIKTLINJE om Fetma och övervikt - behandling i primärvården)

**Orlistat** förhindrar nedbrytningen av fett i kosten och är indicerat för behandling av fetma (BMI ≥30 kg/m^2^) eller övervikt (BMI ≥28 kg/m^2^) med samtidiga riskfaktorer. Behandling med orlistat skall alltid ordineras i kombination med energireducerad kost med begränsat intag av fett (enligt ovan). Följ upp behandlingen efter 4 och 12 veckor. Normaldosering 120 mg x 3. Orlistat kan förskrivas på recept eller köpas receptfritt i en lägre dosering 60 mg x 3.

**Mysimba®** dämpar aptiten och kan användas som tillägg till behandling med energireducerad kost och ökad fysisk aktivitet för viktminskningsbehandling hos personer ≥ 18 år med BMI ≥ 30 kg/m^2^ eller BMI ≥ 27 kg/m^2^ med en eller flera viktrelaterad samsjuklighet som typ 2-diabetes, dyslipidemi eller kontrollerad hypertoni. Dosen ökas successivt från 1 x 1 till 2 x 2 tabletter under 4 veckor. Behandlingen ska avbrytas efter 16 veckor om patienten inte har tappat minst 5% av sin initiala kroppsvikt. Behovet av fortsatt behandling ska utvärderas med intervall på̊ 16 veckor och årligen. Vanliga biverkningar är sömnlöshet, ångest, huvudvärk, bukbesvär, led- och muskelvärk. För användning i patientgrupper som äldre eller personer med olika sjuklighet eller behandlingar, se FASS. Mysimba® ryms inte inom läkemedelsförmånen (juli 2019), varför kostnaden för patienten själv blir cirka 1000 kr/månad.

**Saxenda®** är en GLP-1-receptoragonist verkar bland annat genom att dämpa aptiten, och är indicerat som ett komplement till energireducerad kost och ökad fysisk aktivitet för viktkontroll hos vuxna patienter med BMI ≥30 kg/m^2^ eller BMI ≥27 kg/m^2^ med viktrelaterad samsjuklighet som prediabetes, typ 2-diabetes, hypertoni, dyslipidemi eller obstruktiv sömnapné. Startdosen är 0,6 mg som injiceras subkutant en gång dagligen. Dosen bör ökas till 3,0 mg en gång dagligen i steg om 0,6 mg med minst en veckas intervall. Om upptrappningen till nästa dosnivå inte tolereras två veckor i följd ska avbrytande av behandlingen övervägas. Behandling med 3,0 mg Saxenda® dagligen ska avbrytas efter 12 veckor om patienten inte har förlorat minst 5% av sin initiala kroppsvikt. De vanligaste biverkningarna är illamående eller gastrointestinala besvär. Saxenda® ryms inte inom läkemedelsförmånen (juli 2019), varför kostnaden för patienten själv blir cirka 2500 kr/månad.

**Diabetesläkemedel**

En andel av personer med övervikt och fetma har typ 2-diabetes. Grunden för dess behandling är viktnedgång genom kost-förändringar och förbättrad livsstil, inklusive ökad fysisk aktivitet. Metformin, som ska erbjudas alla personer med typ 2-diabetes om inte begränsande biverkningar eller kontraindikationer föreligger, anses viktneutralt. DPP-4-hämmare är viktneutrala, medan sulfonureider, thiazolidinedioner (pioglitazon) och insulinbehandling generellt ökar vikten. GLP-1-receptoragonister och SGLT-2-hämmare sänker i genomsnitt vikten, och har visat sig minska risken för hjärt- och kärlsjukdom och bevara njurfunktionen vid typ 2-diabetes och hög kardiovaskulär risk. Se Läkemedelsbehandling för glukoskontroll vid typ 2-diabetes. respektive FASS för fullständig information.

# **Avsnitt 6**

# **Riskbruk av alkohol**

## Definition

Riskabla alkoholvanor kan föreligga utifrån riskskalan AUDIT-C vid 4 poäng för kvinnor och 5 poäng för män.

I de vetenskapliga studier som ligger till grund för riktlinjernas rekommendationer ingår dock inte personer med alkoholberoende och de omfattas därför inte heller av rekommendationerna.

Enligt Socialstyrelsen:

- Vid bedömning av riskbeteende utgå från resultat av bedömningsinstrumenten, patientens hälso- och sjukdomstillstånd och samtal med patienten.
- För vissa sjukdomstillstånd är de allmänna råden inte förenliga med de sjukdomsanpassade råden.
- Patienten ska erbjudas råd och stöd utifrån sin situation och sitt individuella behov.
- Råd och information om riskbeteende bör vara kopplat till patientens hälsotillstånd och sjukdom samt till de vinster patienten gör med att sluta med sin ohälsosamma levnadsvana.

## Svårighetsgrad

Måttlig till stor.

## Föreslagen åtgärd

Rådgivande samtal. Se länken nedan! (OBS! Glöm inte grundprincipen i avsnitt 2 på sida 4!).

Bilagor: 4 och 5.

OBS! Bland personer med en alkoholkonsumtion som motsvarar ett riskbruk kan det finnas personer som redan har utvecklat ett missbruk eller beroende. I de vetenskapliga studier som ligger till grund för riktlinjernas rekommendationer ingår dock inte personer med alkoholberoende och de omfattas därför inte heller av rekommendationerna. Rekommendationer om vård och behandling vid alkoholberoende finns i stället i Socialstyrelsens Nationella riktlinjer för vård och stöd vid missbruk och beroende.

## Länkar om riskbruk av alkohol

1. Vårdgivarwebben

<https://www.vgregion.se/halsa-och-vard/vardgivarwebben/vardriktlinjer/levnadsvanor-och-sjukdomsforebyggande-metoder/>alkohol/

Här finns en mer detaljerad beskrivning av arbetet med levnadsvanan Alkohol.

1. Hälsocoach online

<https://www.vgregion.se/halsa-och-vard/levnadsvanor/halsocoach-online/>

# **Avsnitt 7**

# **Otillräcklig fysisk aktivitet**

## Definition

Otillräcklig fysisk aktivitet definieras som en nivå av fysisk aktivitet som inte är tillräcklig för att förbättra den kardiometabola profilen eller förebygga kardiometabol sjukdom.
Även om individens nivå av fysisk aktivitet uppfyller de rekommenderade riktlinjerna för den allmänna befolkningen (enligt definitionen nedan), kan det finnas behov av att höja intensiteten eller förlänga varaktigheten på den fysiska aktiviteten för att ytterligare förbättra den kardiometabola profilen eller förebygga sjukdom.

>150 min/vecka på minst måttlig ansträngningsnivå

*eller* >75 min/vecka på hög ansträngningsnivå

*eller* vid kombination (sammanlagd score) ≥150 min/vecka

Väg in patientens hälso- och sjukdomstillstånd och patientsamtalet i den sammantagna bedömningen. Ta hänsyn till om patienten ska vara fysiskt aktiv i sjukdomsförebyggande eller sjukdomsbehandlande syfte. Vid vissa sjukdomstillstånd är de allmänna råden inte förenliga med de sjukdomsanpassade råden.

Längre tid i stillasittande är kopplat till ohälsa och förtida död. Positiva effekter av att minska tid i stillasittande verkar vara störst för de med låg fysisk aktivitetsgrad. Rekommendationen är att undvika långvarigt stillasittande så mycket som möjligt, inga gränsvärden kan ännu anges vid bedömning utifrån frågorna om stillasittande.

Väg in patientens hälso- och sjukdomstillstånd och patientsamtalet i den sammantagna bedömningen. Vid vissa sjukdomstillstånd är de allmänna råden inte förenliga med de sjukdomsanpassade råden.

Enligt Socialstyrelsen:

- Vid bedömning av riskbeteende utgå från resultat av bedömningsinstrumenten, patientens hälso- och sjukdomstillstånd och samtal med patienten.
- Patienten ska erbjudas råd och stöd utifrån sin situation och sitt individuella behov.
- Råd och information om riskbeteende bör vara kopplat till patientens hälsotillstånd och sjukdom samt till de vinster patienten gör med att sluta med sin ohälsosamma levnadsvana.

## Svårighetsgrad

Måttlig till stor.

## Föreslagen åtgärd

Rådgivande samtal med eller utan tillägg av skriftlig ordination av fysisk aktivitet eller aktivitetsmätare. (OBS! Glöm inte grundprincipen i avsnitt 2 på sida 4!).

Individer med kroniska sjukdomstillstånd eller funktionshinder, som inte kan nå upp till rekommendationerna, bör vara så aktiva som tillståndet medger. Valet av aktiviteter kan behöva anpassas till tillståndet. I dessa fall kan specifika rekommendationer erhållas i FYSS (www.fyss.se), en handbok om fysisk aktivitet i sjukdomsprevention och sjukdomsbehandling.

## Länkar om rådgivning och hänvisning till fysisk aktivitet

1. Vårdgivarwebbens sidor med information, länkar och material att använda:

<https://www.vgregion.se/halsa-och-vard/vardgivarwebben/vardriktlinjer/levnadsvanor-och-sjukdomsforebyggande-metoder/fysisk-aktivitet/>

<https://www.vgregion.se/halsa-och-vard/vardgivarwebben/vardriktlinjer/levnadsvanor-och-sjukdomsforebyggande-metoder/fysisk-aktivitet/hanvisa-patienter-vidare/>

Vårdgivarwebbens sammanställning av länkar till verksamheterna som beskrivs nedan.

1. Aktivitetskatalogen, ges ut årligen av Centrum för fysisk aktivitet

<https://www.vgregion.se/halsa-och-vard/levnadsvanor/aktivitetskatalog-far>

Överskådlig sammanställning av många olika aktörer, såsom idrottsföreningar, motionsanläggningar och träffpunkter för seniorer, m.m. Finns även att beställa som fysisk katalog.

1. Kort beskrivning av verksamheter att hänvisa till:

Hälsolots Majorna-Linné, Hälsoteket Angered, Hälsoteket Väster, Hälsoteket Örgryte-Härlanda, Hälsoteket Östra Göteborg, Livskraften Härryda och Hälsodisken Hisingen

<https://www.halsolots.se/>

Erbjuder motionsaktiviteter m.m. Nästan alltid kostnadsfritt och drop in.

1. Friskvårdsklubben – ideell förening som riktar sig till målgruppen med personer som har eller haft psykisk ohälsa. Gymträning, olika sporter och aktiviteter.

<https://www.friskvardsklubben.se/>

Passar för individer som vill träna tillsammans med andra som har någon form av psykisk ohälsa. Medlemsavgift och eventuella andra avgifter är mycket låga jämfört med träningskort på motionsanläggningar.

1. FaR-mottagningar

<https://www.vgregion.se/halsa-och-vard/levnadsvanor/aktivitetskatalog-far/far-mottagning/>

Dessa mottagningar kan vi hänvisa patienter som fått Fysisk aktivitet på recept (FaR). Här finns fysioterapeut på plats och patienten kan boka tid för vägledning i val av aktiviteter, utformning av träningsprogram, samt stöttning att komma i gång. Det finns möjlighet till uppföljande stöd i 6 månader och även återkoppling till oss efter avslutad insats om patienten medger det. Kostnadsfritt.

1. Hälsocoach online

<https://www.vgregion.se/halsa-och-vard/levnadsvanor/halsocoach-online/>

1. Aktiv, via Finsam

<https://finsamgoteborg.se/insatser/information-om-insatserna/#aktiv-2>

Målgruppen för Finsam Göteborg är personer i åldrarna 16–64 som har kontakt med minst två av följande: FK, AF, VGR, Göteborgs stad, och har behov av samordnat stöd för att nå eller närma sig arbete eller studier.

”Vi finns för dig som:

Är 16–64 år och bor i Göteborg

Har psykisk och/eller fysisk ohälsa som utgör hinder att arbeta eller studera

Är eller riskerar att bli sjukskriven och bedöms att genom träning kunna korta av eller förebygga sjukskrivning

För närvarande inte arbetar och har behov av att stärka hälsan som en del av din planering mot arbete eller studier

Aktiv erbjuder gymträning och hälsofrämjande stöd under en begränsad tidsperiod. Syftet är att öka arbetsförmågan genom att förbättra den fysiska och psykiska hälsan med hjälp av att komma i gång med ett individuellt anpassat träningsupplägg.”

1. 1177 Träning och fysisk hälsa

<https://www.1177.se/Vastra-Gotaland/liv--halsa/fysisk-aktivitet-och-traning/>

Sida med information, tips, och filmer.

1. Göteborgs stads sida med tips om fysisk aktivitet.

<https://goteborg.se>

1. Länkar om Fysisk aktivitet på recept, FaR

Fysisk aktivitet kan ordineras av all legitimerad personal som har tillräcklig kunskap om metoden FaR. Det finns kostnadsfria interna utbildningar via Centrum för fysisk aktivitet.

På Vårdgivarwebben och via Hälsofrämjande sjukhus hittar du information, material och lathundar. Här är förslag på några användbara länkar för att komma i gång:

Vårdgivarwebbens information om FaR

<https://www.vgregion.se/fysiskaktivitet>

Uppföljning

FaR-uppföljningen anpassas till patientens behov. Tätare kontakt vid mindre grad av självständighet, glesare kontakt om det finns starka resurser att göra förändringen på egen hand. Som glesast ska uppföljning göras var 6:e månad.

Om patienten avslutas på mottagningen ska ansvaret för FaR-uppföljning meddelas den aktuella mottagningen/vårdcentralen.

# **Avsnitt 8**

# **Bilagor**

**Bilaga 1. Flödesschema (Se originalet!)**


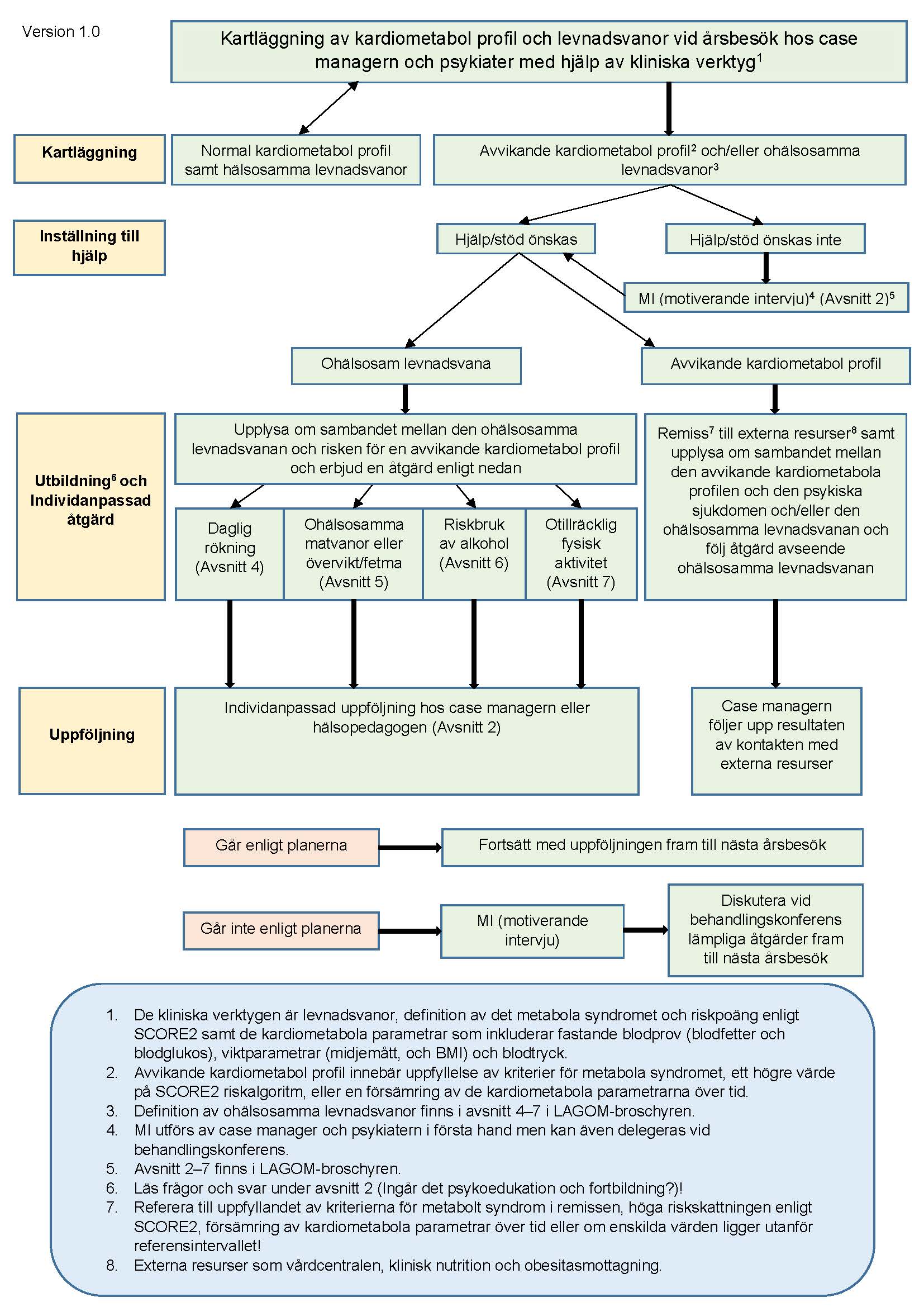


**Bilaga 2. Matvanor och kostindex**

**Bilaga 3. Obligatorisk bilaga till vårdbegäran/remiss för fetmabehandling**

**Bilaga 4. AUDIT-C**

**Bilaga 5. Tolkningsstöd för frågor om alkohol enligt AUDIT-C**


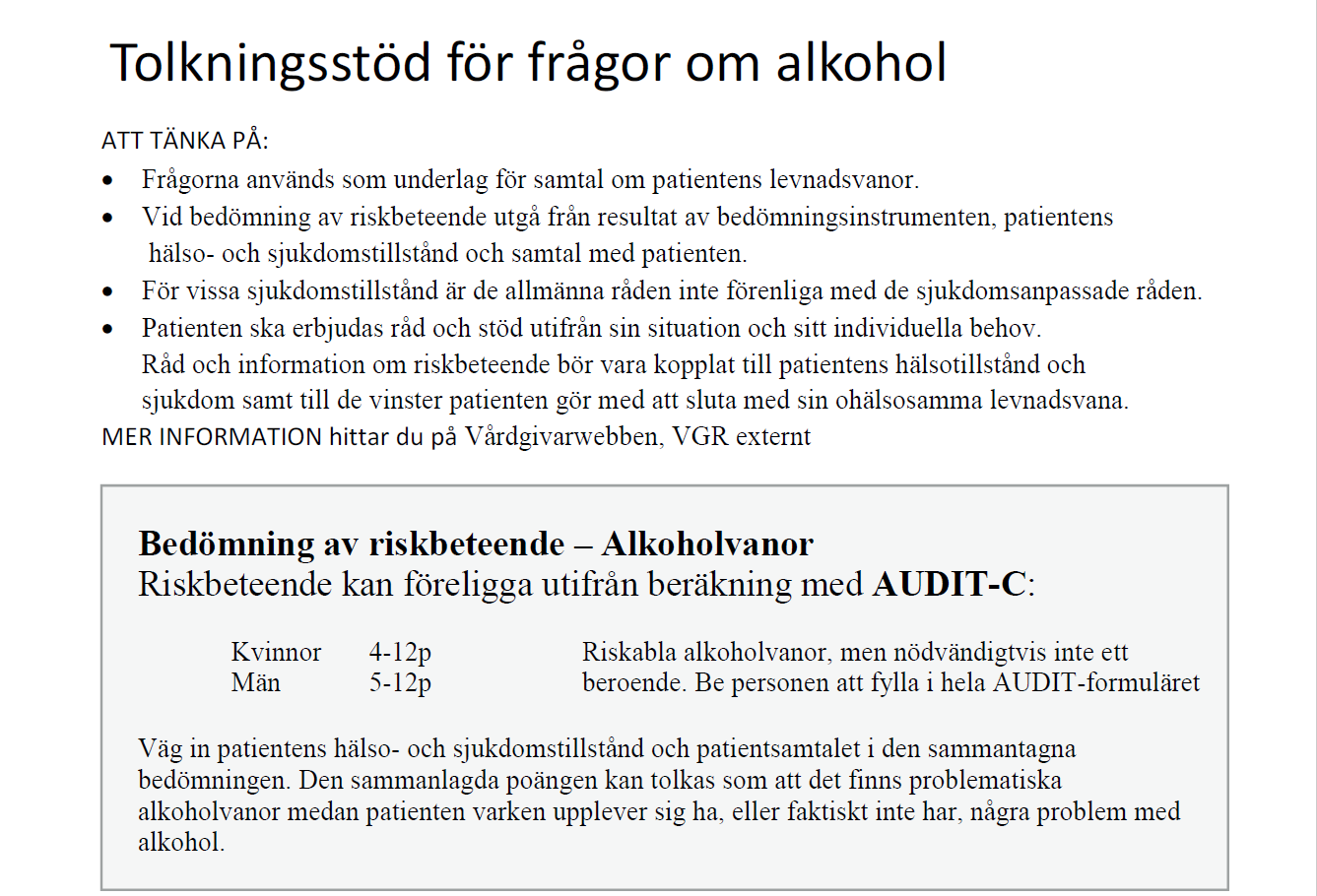

Supplement: Supplementary file 2 — Supplementary Material 2 [file 12888_2026_8315_MOESM2_ESM.docx]
